# Recent advancements in artificial intelligence‐powered cancer prediction from oral microbiome

**DOI:** 10.1111/prd.70000

**Published:** 2025-09-11

**Authors:** Negin Soghli, Aminollah Khormali, Darius Mahboubi, Aimin Peng, Patricia A. Miguez

**Affiliations:** ^1^ Department of Biomedical Sciences, Adams School of Dentistry The University of North Carolina at Chapel Hill Chapel Hill North Carolina USA; ^2^ Adams School of Dentistry The University of North Carolina at Chapel Hill Chapel Hill North Carolina USA; ^3^ Lineberger Comprehensive Cancer Center The University of North Carolina at Chapel Hill Chapel Hill North Carolina USA; ^4^ Department of Periodontology, Endodontics and Dental Hygiene, Adams School of Dentistry The University of North Carolina at Chapel Hill Chapel Hill North Carolina USA

**Keywords:** Keywordsartificial inteligence, cancer prediction, machine learning, oral microbiome, periodontitis

## Abstract

Oral cancer is a major global health burden, ranking sixth in prevalence, with oral squamous cell carcinoma (OSCC) being the most common type. Importantly, OSCC is often diagnosed at late stages, underscoring the need for innovative methods for early detection. The oral microbiome, an active microbial community within the oral cavity, holds promise as a biomarker for the prediction and progression of cancer. Emerging computational techniques in the artificial intelligence (AI) field have enabled the analysis of complex microbiome data sets to unravel the association between oral microbiome composition and oral cancer. This review provides a comprehensive overview of learning‐based algorithms applied to oral microbiome data for cancer prediction. In particular, this work discusses how typical machine learning (ML) algorithms, such as logistic regression, random forests, and artificial neural networks, identify the unique microbial patterns associated with oral cancer and other malignancies. A search was conducted in Pubmed covering a 10‐year period. The goal was to identify previous studies focused on the role of the oral microbiome in oral cancer prediction using AI‐powered tools. The search strategy identified 3382 records in total, of which 44 studies met the inclusion criteria. While AI has shown a transformative power in understanding and revealing the oral microbiome's role in cancer studies, its application in clinical settings requires further efforts on standardization of protocols, curation of diverse cohorts, and validation through large‐scale multi‐centric and longitudinal studies. The integration of AI with oral microbiome analysis holds significant promise for improving early detection, risk stratification, and personalized treatment strategies for OSCC. By identifying unique microbial patterns associated with cancer, AI‐driven models offer a noninvasive, cost‐effective tool to predict disease progression and guide clinical decision‐making. However, translating these advancements into routine clinical practice requires standardized protocols, diverse patient cohorts, and validation through large‐scale, longitudinal studies. Once implemented, this approach could transform oral cancer management, enabling timely interventions and improving patient outcomes.

## 1 INTRODUCTION

Oral squamous cell carcinoma (OSCC) is a malignancy originating in the stratified squamous epithelium of the mouth. OSCC influences different anatomical subsites such as lips, tongue, gingiva, buccal mucosa, floor of the mouth, hard palate, and oropharynx.^1^ OSCC, together with oropharyngeal squamous cell carcinoma (OPSCC), constitutes the majority of head and neck squamous cell carcinoma (HNSCC) cases.^2^ Oral cancer globally accounted for 389 846 new cases and 188 438 deaths in 2022, showing an increase in the number of new cases and deaths compared to 2020 (377 713 new cases and 177 757 deaths in 2020).^3^, ^4^ It is projected that in 2025, approximately 59 660 new cases of OSCC will be diagnosed in the United States, with luminal percentages of about 2.9% of total new cancer diagnoses.^5^ Currently, the mainstay of treatment for OSCC is a multimodal approach whereby surgical resection of the primary tumor is combined with radiation therapy and chemotherapy, particularly for advanced diseases with high‐risk features or for those with distant metastasis.^6^ Despite advances in surgical techniques, radiation modalities, and chemotherapeutic regimens over the last several decades, the 5‐year overall survival rate for OSCC has shown inadequate improvement, especially for advanced‐stage patients.^7^ This challenge persists and requires more refined tools for detection and diagnosis and the development of alternative therapeutic interventions to overcome resistance to treatment, thereby improving patient outcomes. The tumor microenvironment, which connects tumor cells, stromal cells, immune cells, and the resident microbiome, is increasingly viewed as an essential determinant of cancer recurrence, progression, and response to treatment, which would be one of the hallmarks of cancer as described by Hanahan and Weinberg.^8^

The human microbiome is a complex and dynamic ecosystem of microorganisms, including bacteria, viruses, fungi, archaea and protozoa that inhabit various anatomical niches in the human body.^9^ This diverse microbial community interacts with the host through a complex network of molecular signaling pathways and influences many physiological processes including immune system development and function, nutrient metabolism and regulation of inflammatory responses with huge implications for human health and disease susceptibility including cancer.^10^ The advent of high‐throughput next generation‐sequencing (NGS) technologies^11^ such as 16S rRNA gene sequencing^12^ for bacterial community profiling, whole‐genome shotgun metagenomic sequencing (for comprehensive genomic analysis of the microbiome) and metatranscriptomics (for functional activity of the microbial community)^13^ has revolutionized the study of microbiome composition, structure and functional potential. These advancements coupled with omics approaches including metabolomics (for small molecules produced by microbial metabolism) and proteomics (for microbial protein expression)^13^ and large‐scale collaborative initiatives such as the Human Microbiome Project^14^ has greatly expanded the understanding of the microbiome's multiple roles in both health and disease. This has allowed us to focus on densely populated microbial niches in the human body including the oral cavity, respiratory tract, gastrointestinal tract, and skin and urogenital tract.^15^, ^16^, ^17^ Many studies have shown a close and often site‐specific association between the composition and function of the resident microbiota and the development and progression of various disease states. For example, the gut microbiota has been implicated in the pathogenesis of various gastrointestinal disorders including inflammatory bowel disease and colorectal cancer while the oral microbiome is now recognized as a key player in oral health and maintenance of oral homeostasis and development of various oral diseases including periodontal diseases and oral cancers.^18^, ^19^

Traditionally, the oral microbiota has been studied in the context of nonneoplastic diseases such as periodontitis, gingivitis, and dental caries.^20^, ^21^, ^22^ However, there is a growing interest in the role of oral microbiota in oral cancer, especially OSCC.^23^, ^24^ Alterations in oral microbiota community composition have been related to well‐known risk factors for oral cancer, including alcohol consumption, tobacco smoking, and betel nut chewing. Contreras et al.^25^ examined the global impact of seven oncoviruses, which cause 20% of human cancers, by detailing their primary carcinogenic mechanisms, such as cellular transformation, angiogenesis, and inflammation, and exploring the potential for SARS‐CoV‐2 to reactivate latent oncoviruses. They ultimately highlighted the promise of oncovirus vaccines and the need for further research into host and environmental cofactors. Analogous microbial changes have also been observed in precancerous oral lesions such as leukoplakia and lichen planus.^26^ More recently, studies have shown significant differences in the microbiota of tumor tissue, adjacent nonneoplastic tissue, and saliva in patients with OSCC.^23^, ^27^, ^28^ Such findings suggest the potential use of the oral microbiome as a prognostic marker in oral cancers,^27^, ^28^ emphasizing a strong link with OSCC. Although in vitro studies present compelling evidence for the carcinogenicity of *Fusobacterium nucleatum* and *Porphyromonas gingivalis*, clinical investigations on the oral microbiome and oral cancer often yield inconsistent results, possibly attributable to methodological variations or the phenomenon of functional redundancy. Consequently, rather than specific microbial species acting as primary etiological agents in OSCC, it is the dysregulated function of the tumor microenvironment's “passenger” microbiome that is hypothesized to drive tumor progression through the sustenance of chronic inflammation.^29^

Predictive machine learning (ML) models have become essential for analyzing complex biological data as they can detect patterns that would otherwise go unnoticed. Hence, they are used in healthcare applications like imaging analysis, electronic health record management, and genetic studies.^30^, ^31^ ML algorithms can process complex microbial signatures, making it a useful tool in microbiome research, especially for host–microbe interactions.^32^ While early studies focused on identifying the association between gut microbiome and specific diseases like colorectal cancer using ML,^33^ current applications of ML are biomarker discovery, disease progression, and therapeutic response through analysis of tumor, gut, and oral microbiomes across the spectrum of cancers.^34^, ^35^, ^36^ The scope of this review is to identify the relationship between oral microbiome and oral cancer with a focus on the role of AI in identifying this association.

## 2 MATERIALS AND METHODS

A literature search was performed in August 2024 on studies published in the previous 10 years and indexed in PubMed. The following search terms were used: *microbiota AND oral cancer AND artificial intelligence* OR *microbiota AND oral squamous cell carcinoma AND artificial intelligence* OR *microbiota AND head and neck cancer AND artificial intelligence*. All MeSH terms used for the search in PubMed are listed in the Appendix S1. A total number of 3382 papers were found during the search. Two reviewers independently selected the articles meeting the inclusion criteria. Reviewer number 1 (N.S.) reviewed first, followed by reviewer number 2 (A.K.), which was done independently. If necessary, reviewer number 3 (D.M.) reviewed any disparities between the two initial reviews. Two independent reviewers selected the articles based on the inclusion/exclusion criteria applied first to the title/abstract level and then to the full‐text article. The inclusion criteria were (1) studies investigating the role of the oral microbiome in oral cancer (particularly OSCC) and its association with cancer prediction, progression, or treatment outcomes; (2) studies utilizing artificial intelligence (AI) or ML techniques to analyze oral microbiome data; (3) cohort studies, case–control studies, and cross‐sectional studies involving high‐throughput sequencing data (e.g., 16S rRNA, metagenomics) of the oral microbiome; (4) studies published in English; and (5) studies published within the last 10 years (2014–2024) to ensure relevance to current AI and microbiome research advancements. The exclusion criteria were as follows: (1) studies not related to oral cancer (e.g., other types of cancer or noncancerous oral diseases) or those that do not investigate the oral microbiome; (2) review articles including both narrative and systematic reviews; (3) case reports, editorials, opinion pieces, or studies with insufficient methodological detail; and (4) duplicate studies.

The found articles were screened in two stages, including title and abstract screening and full‐text review. In the first phase, studies were screened based on their titles and abstracts to assess their relevance to the reviewer's objectives. Then, potentially relevant studies were subjected to full‐text review to ensure the studies met the inclusion criteria. Eligible studies (44 papers at the final stage) were used for extracting the data using a standardized template, including the following items: (1) study characteristics (author, year, country, study design), (2) sample size and cohort demographics, (3) microbiome sequencing and bioinformatic methods, (4) AI/ML algorithms used, (5) prognostic values, and (6) outcomes. Data extraction had been completed independently, and the extractors crosschecked the data for consensus. In the case of any ambiguous or missing information, we contacted the corresponding author for key information.

## 3 ORAL MICROBIOTA AND ORAL CANCER

**FIGURE 1:**
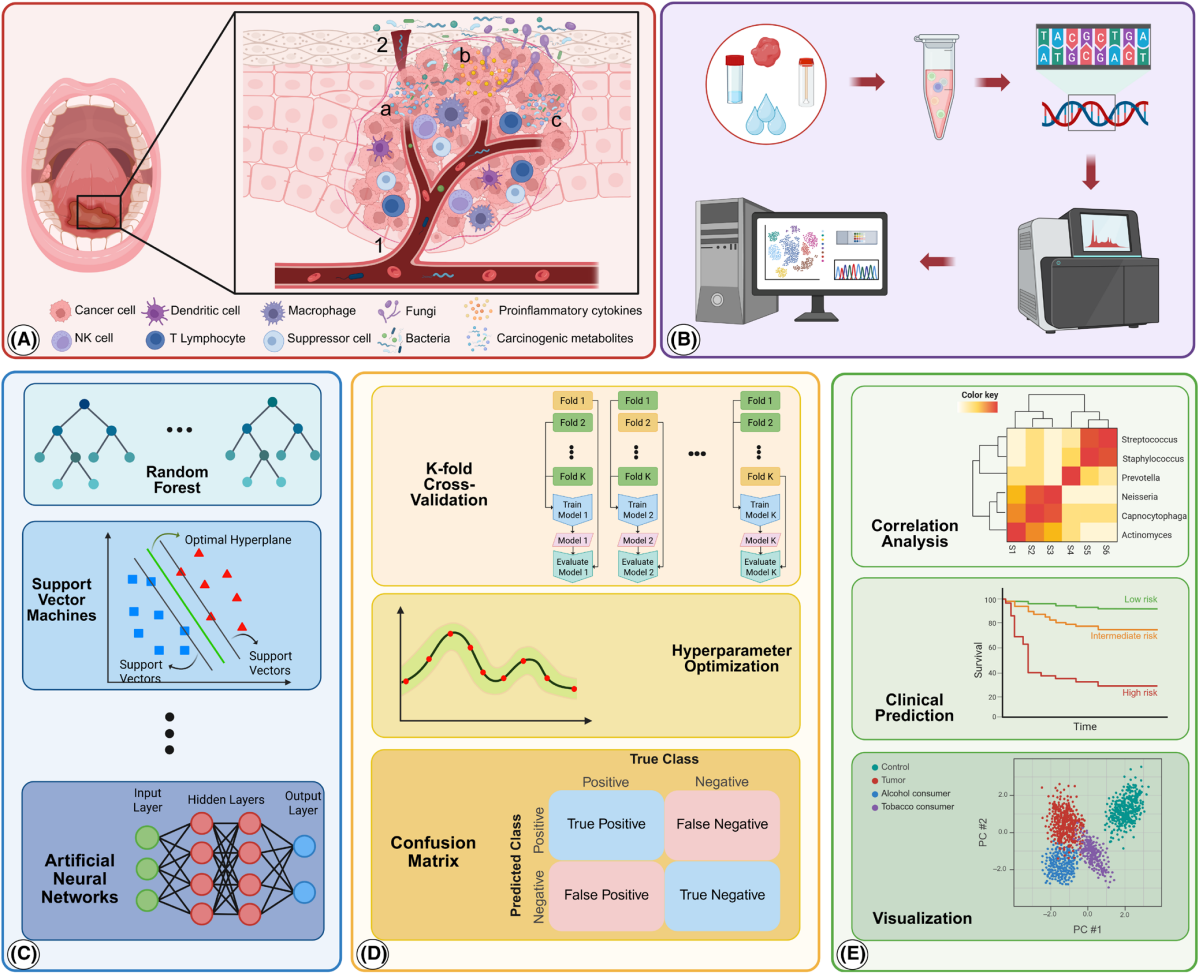
A schematic overview of AI‐powered cancer prediction from oral microbiome data. (A) Interactions between microorganisms and the tumor microenvironment in oral squamous cell carcinoma: This panel illustrates how microorganisms influence the development and progression of OSCC. Bacteria and fungi can enter the tumor site through the bloodstream (1) or from nearby tissues like periodontal pockets (2). Their presence can contribute to a cancerous environment by producing harmful metabolites (a), triggering inflammation (b), and disrupting the balance between cell growth and cell death (c). The interaction between immune cells (NK cells, macrophages, dendritic cells, and lymphocytes) further shapes the tumor microenvironment and promotes cancer development. (B) Data Collection and High‐throughput Sequencing Pipelines: This panel depicts the process of collecting and analyzing microbiome samples (from oral tissues or fluids) using high‐throughput sequencing technologies. This analysis provides detailed information about the microbial communities present. (C) Machine Learning (ML) for Cancer Prediction: This panel explains how ML algorithms (e.g., random forest, support vector machines, artificial neural networks) are used to analyze microbiome data and predict cancer outcomes (please see Section 5 for more information about AI algorithms). (D) ML Model Training and Evaluation: This panel highlights the importance of ensuring the accuracy and reliability of the ML models. Techniques used include k‐fold crossvalidation to prevent overfitting, hyperparameter optimization to tune model parameters, and performance evaluation using confusion matrices to assess the model's ability to correctly classify cancer and noncancer cases. (E) Data Visualization and Correlation Analysis: This panel describes how data visualization techniques (e.g., principal component analysis, survival time, hierarchical clustering) are used to identify patterns in the microbiome data. By analyzing these patterns, researchers can identify specific microbial signatures associated with OSCC progression (Created with BioRender.com). OSCC, oral squamous cell carcinoma.

The human oral cavity is home to a diverse and complex community of microbes that is associated with oral and overall health. Recently, it has been shown that disruptions to this delicate balance of microbes, known as dysbiosis, can contribute to the development and progression of various cancers, including OSCC.^37^ The oral microbiota, which is made up of bacteria, viruses, fungi, and other microorganisms, interacts with host tissues and immune responses in ways that can influence carcinogenesis.^38^ As we learn more about the human microbiome, a growing body of literature is now linking specific oral pathogens and microbial community structures to increased cancer risk and poorer prognosis.^39^ Environmental and lifestyle factors, such as tobacco use, alcohol consumption, poor oral hygiene, and diet can shift the oral microbiome, allowing pathogenic bacteria to overgrow and reduce microbial diversity. This dysbiotic environment, as shown in panel (A) of Figure 1, allows microbes to get into the tumor site through the bloodstream or from nearby tissues like periodontal pockets. These microbes produce toxic metabolites, trigger inflammation, and disrupt the balance of cell growth and cell death. The interaction between immune cells, including macrophages, dendritic cells, T lymphocytes, and natural killer cells, in this altered environment further promotes cancer development. These immune cells, while essential for host defense, can become dysregulated in the tumor microenvironment and lead to immunosuppression and tumor escape. These changes create a dysbiotic environment that promotes chronic inflammation and activates oncogenic pathways including TNFAIP8 and IL‐6/STAT3, which are known to support inflammatory states that are favorable to cancer development.^40^ The oral microbiome contributes to carcinogenesis through mechanisms like epithelial barrier dysfunction, chronic inflammation, and epigenetic modulation, with specific bacteria like *Treponema denticola* and *F. nucleatum* promoting cancer progression via pathways such as TLR2/MyD88 and E‐cadherin/β‐catenin signaling.^41^, ^42^ The emerging link between microbial shifts and oral cancer risk provides a framework for future research into the specific risk factors and mechanisms of oral carcinogenesis.

Involving the multi‐step process shown in Figure 1, AI investigates the connection between the oral microbiome and oral malignancy. The oral microbiome characterization starts from the collection of oral samples, such as saliva, rinses, swabs, or biopsies. Following that, NGS technologies are employed to generate comprehensive data on the microbial composition, including taxonomic profiles and functional potential. To construct a descriptive profile of the microbial composition from those data, further analysis is performed using a variety of ML algorithms, for example, random forest,^43^ support vector machines,^44^ and artificial neural networks,^45^ to discover patterns within microbiome data and predict cancer outcomes. Thus, with a better understanding of the sophisticated patterns and relationships within this data of the microbiome, these algorithms are capable of predicting cancer outcomes. However, to ensure the reliability and accuracy of the predictions for cancer outcomes, rigorous training and testing of these algorithms are performed using techniques such as k‐fold cross‐validation^46^ and hyperparameter optimization.^47^ The predictive framework, together with the generated prediction, is then used for further investigation and exploration using visualization techniques such as principal component analysis (PCA),^48^ survival analysis, and hierarchical clustering.^49^ While some specific bacterial taxa and clinical variables are examined through correlation analysis, others, like Kaplan–Meier survival curves,^50^ show the prognostic utility of the identified microbiome signatures. Eventually, two‐dimensional PCA shows well‐defined clusters of samples within which they may be grouped according to their microbial composition and hence present a possible association with clinical outcomes.

### 3.1 Oral cancer risk factors

OSCC is the most common form of oral malignant disease, which originates predominantly from the epithelial tissue of the mucosa lining the oral cavity.^1^ The mucous membrane lining the oral cavity has two main components: a stratified squamous epithelium, which may keratinize, and the lamina propria.^51^ Studies have clearly shown the association of oral cancers with certain periodontal pathogens, namely *P. gingivalis*, *F. nucleatum*, and *T. denticola*.^52^ Furthermore, increased prevalence of certain proinflammatory bacteria, specifically *Prevotella*, *Capnocytophaga*, and *Fusobacterium*, was reported in association with smokeless tobacco usage.^53^, ^54^ Beyond the influence of individual bacterial species, both fungal species, such as Candida, and high‐risk human papillomavirus (HPV) genotypes, particularly HPV16, have been associated with oral cancer risk.^55^ Moreover, polymicrobial synergy and dysbiosis resulting from inter‐microbial interactions within the oral microbiome are implicated in the pathogenesis and progression of oral cancer.^56^, ^57^ All these observations point toward the complexity of the interplay of the oral microbiome in relation to oral carcinogenesis.

As specified in epidemiological research, oral cancer is correlated with various regional burdens; it has a very high toll in South and Southeast Asia, especially in India, accounting for one‐third of the entire burden in the world.^58^ Health socioeconomic determinants induce a different amount of burden globally, while almost all developing countries experience particularly high burdens because of a lack of access to health care infrastructure with preventive measures.^59^ Despite improved access to treatment modalities and early diagnostic capabilities in developed nations, the prognosis remains suboptimal, with a five‐year survival rate of only 65% reported in the United States.^58^ Moreover, high alcohol consumption, in particular, has been linked to an increased risk of oral cancer in a dose‐dependent manner. Heavy drinkers consuming 4–5 drinks on a daily basis have a 2–3 times higher risk of oral cancer compared to nondrinkers. The major reason for this observation has been attributed to the ethanol present in alcoholic drinks, which imposes mutagenic effects on the DNA through its metabolism byproduct, acetaldehyde.^60^, ^61^, ^62^ A report by the International Agency for Research on Cancer (IACR) highlighted that beers contain nitrosamines, which are additional carcinogens, and it might explain the increased risk of colorectal cancer in beer drinkers. In addition, the report acknowledges that while wines contain polyphenols, which may have some cardiovascular benefits, these do not outweigh the carcinogenic effects of ethanol. Moreover, the IARC monograph notes that spirits have a higher ethanol concentration, which may contribute to the higher risk per unit of alcohol consumed.^63^ Similarly, smoking is a major etiologic factor for cancers of the lip, oral cavity, and pharynx. Data from the 2019 Global Burden of Disease Study demonstrate that smoking was responsible for 55.8% of other pharyngeal cancer deaths among males and 17.4% among females.^64^ These lifestyle factors not only elevate the risk of carcinogenesis but also induce changes in the oral microbiota, resulting in a reduction of beneficial microbial diversity and an enrichment of pathogenic bacteria. Such a resultant dysbiosis may, therefore, either help or play some role in the pathogenesis and development of some disease states and cancers of the oral cavity. For this reason, the adoption of healthy lifestyles, which will include moderation in alcohol consumption, avoidance of tobacco products, and adequate oral hygiene practice, is necessary for maintaining appropriate and balanced microbiota and reducing diseases. These disparities do point to the need for targeted preventive strategies and public health interventions.

The oral microbiome indeed contributes to the endogenous immunity that could lead to triggering and propagating the carcinogenic process in oral mucosa through modulating the immune responses and causing inflammation.^65^, ^66^ This means that the state of the oral microbiome or change within it could preferably cause an increase in *Streptococcus*, *Abiotrophia*, and *Leuconostoc* and a decrease in *Prevotella*, *Haemophilus*, and *Neisseria* that might place more risk for malignancy development.^40^ These shifts in microbial composition might modify the levels of short‐chain fatty acids (SCFAs) and would contribute to lowering SCFA levels as well as down‐regulation of free fatty acid receptor 2 expression, both important for the maintenance of the anti‐inflammatory state. Dysbiosis interferes with the immune regulations and provokes the activation of pro‐inflammatory pathways such as TNFAIP8 and IL‐6/STAT3, which are known to favor inflammatory status to possibly support cancer development.^40^

### 3.2 Oral microbiota dysbiosis and oral cancer progression

**FIGURE 2:**
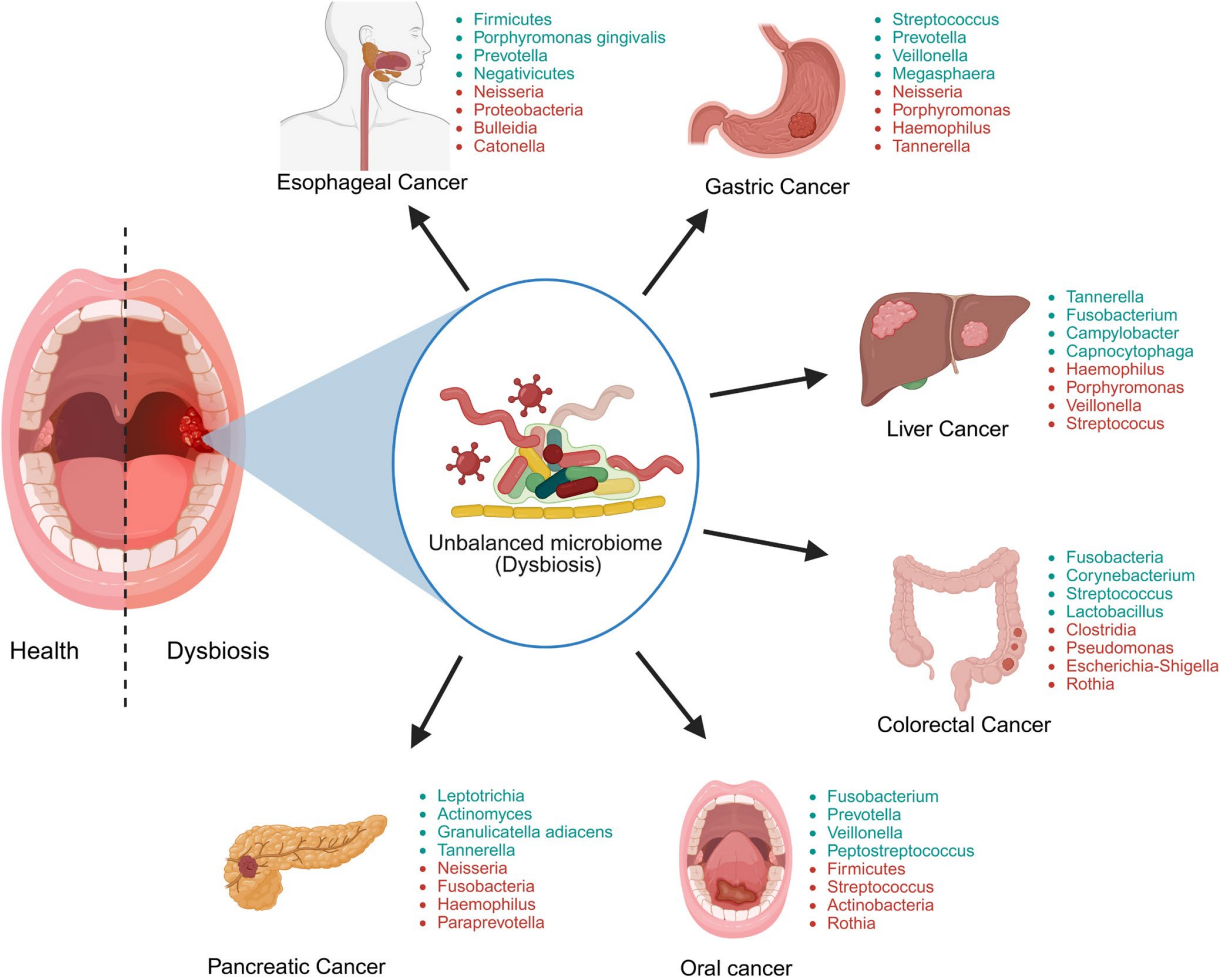
The interplay of dysbiosis in oral microbiota and cancer development. This figure depicts the association between oral microbiome dysbiosis and the development of various cancers in the digestive tract. Starting from the oral cavity, dysbiosis can contribute to oral cancer. Furthermore, dysbiosis can extend to other organs, influencing the development of esophageal cancer, gastric cancer, liver cancer, pancreatic cancer, and colorectal cancer. The figure highlights some of the key bacterial genera associated with each cancer type, demonstrating the potential for microbial shifts to contribute to carcinogenesis across different sites within the digestive system (Created with BioRender.com).

Composition changes in the oral microbiota indicative of dysbiosis have been correlated to oral cancers and neoplasms of distant organs, including the esophagus, stomach, liver, pancreas, and colon. Some studies have investigated the current epidemiological evidence, primarily from cohort and case–control studies, supporting a widespread association between periodontitis and various human cancers, including those of the oral cavity, gastrointestinal tract, lung, pancreas, prostate, liver, breast, and ovaries. A current lack of treatment trial evidence to definitively establish periodontitis's causal role in cancer initiation and development.^67^ Certain bacterial genera such as *Fusobacterium*, *Prevotella*, *Streptococcus*, and *Rothia* have been found to be involved in oral cancer while distinct microbial communities have been seen in cancers of these distant sites.^68^ Understanding such variations in the microbiota in human hosts gives essential information regarding local and systemic dysbiosis that could contribute to carcinogenesis, as illustrated in Figure 2. Furthermore, Rynazal et al.^69^ investigated the use of explainable AI for the gut microbiome. Using global explanation methods, studies have linked colorectal cancer to gut microbiome compositions and identified general bacterial biomarkers. However, this study demonstrates that Shapley Additive Explanations (SHAP) can offer a more personalized approach, revealing individual‐specific bacterial biomarkers and even distinguishing CRC subgroups with varying probabilities. Studies on the oral microbiome and oral potentially malignant disorders have given us valuable information on how specific microbial changes affect cancer risk. Notably, microbial changes in the oral cavity have been seen during the transition from healthy to precancerous lesions.^70^ Diverse microbial changes are involved in the early stages of oral cancer development. For example, Lee et al.^71^ have demonstrated a decreased presence of some bacteria, including *Bacillus*, *Parvimonas*, *Peptostreptococcus*, and *Enterococcus*, in dysplastic tissue and, therefore, suggesting a connection to early malignant transformations. Similarly, some studies such as those by Gopinath et al.^72^ have reported an increase in *Megasphaera*, *nonclassified Enterobacteriaceae*, *Prevotella*, and *Salmonella* in leukoplakia, as a form of OPMD, suggesting that these cases have an overlap with the oral cancer profiles. This evidence shows that entire microbial dynamics involve such complex systems. All of these studies are in Table 1.

**TABLE 1:** Characteristics and the various methods used for sample collection and sample processing in each study.

Author (year)	Country	Lesion type(s)	Lesion site(s)	Sampling type	Microbiota type	Microbiota characterization	Cohort size
Warnke‐Sommer et al. (2017)^107^	USA	OC	Oral cavity	Swab samples	Total bacterial spectrum and abundance ratio	Illumina MiSeq targeting V4 variable region	38
Bornigen et al. (2017)^108^	USA	OSCC, OPSCC	Oral cavity, oropharynx	Oral rinse samples	Total bacterial spectrum and abundance ratio	Illumina MiSeq targeting V4 variable region	363
Lee et al. (2017)^71^	Taiwan	OSCC, OPMD	Tongue, floor of the mouth, lip, buccal mucosa, alveolar ridge, hard palate	Unstimulated saliva	Total bacterial spectrum and abundance ratio	PCR primers targeting V3–V4 regions of 16S rRNA gene (515F/806R)	376
Mok et al. (2017)^109^	Malaysia	OSCC, OPMD	Oral cavity	Swab samples	Total bacterial spectrum and abundance ratio	Primer pair D88/E94, targets V6–V9 regions of 16S	27
Wang et al. (2017)^86^	USA	HNSCC	Oral cavity, oropharynx, hypopharynx, larynx	Paired normal and tumor tissues	Total bacterial spectrum and abundance ratio	PCR for V1–V4 hypervariable regions of 16S rRNA	242
Zhao et al. (2017)^110^	China	OSCC	Oral cavity	Swab samples	Total bacterial spectrum and abundance ratio	PCR primers targeting 16S rRNA gene's V3–V4 regions (515F/926R)	80
Hayes et al. (2018)^83^	USA	HNSCC	Oral cavity, pharynx, larynx	Mouth wash samples	Total bacterial spectrum and abundance ratio	PCR primers targeting V3–V4 regions of 16S rRNA gene (347F/803R)	383
Hsiao et al. (2018)^111^	Taiwan	OSCC	Oral cavity	Unstimulated saliva, Blood	Comparative analysis of 20 bacterial species	PCR primers targeting 16S rRNA gene's V3–V4 regions (341F/926R)	289
Lim et al. (2018)^112^	Australia	OCC, OPC	Oral cavity, oropharynx	Oral rinse samples	Total bacterial spectrum and abundance ratio	PCR primers targeting 16S rRNA gene's V3–V4 regions (515F/806R)	83
Perera et al. (2018)^113^	Sri Lanka	OSCC	Tongue, buccal mucosa	Tissue biopsy samples	Total bacterial spectrum and abundance ratio	PCR primers targeting 16S rRNA gene's V1–V3 regions (27FYMF/519R)	52
Vesty et al. (2018)^114^	New Zealand	HNSCC	Oral cavity, left parotid, tonsils	Unstimulated saliva	Total bacterial spectrum and abundance ratio	PCR primers targeting 16S rRNA gene's V1–V3 regions (341F/806R)	30
Yang et al. (2018)^115^	Taiwan	OSCC	Buccal mucosa, tongue, lip, gingiva	Unstimulated saliva	Total bacterial spectrum and abundance ratio	PCR primers targeting 16S rRNA gene's V1–V3 regions (F515/R806)	39
Yang et al. (2018)^74^	Taiwan	OSCC	Tongue, gingiva, floor of the mouth	Oral rinse samples	Total bacterial spectrum and abundance ratio	PCR primers targeting 16S rRNA gene's V1–V3 regions (16SF/16SR)	248
Yost et al. (2018)^84^	USA	OSCC	–	Swab samples	Active microbial communities profiling	cDNA Amplification from mRNA Using Illumina Adapter‐Specific Primers	15
Ganly et al. (2019)^105^	USA	OSCC, OPMD	Oral cavity	Oral rinse samples	Total bacterial spectrum and abundance ratio	PCR primers targeting 16S rRNA gene's V1–V3 regions (347F/803R)	38
Hashimoto et al. (2019)^79^	Japan	OSCC, OPMD	Tongue, gingiva, buccal mucosa	Unstimulated saliva	Total bacterial spectrum and abundance ratio	PCR primers targeting 16S rRNA gene's V1–V3 regions (F515/R806)	16
Robayo et al. (2019)^104^	Colombia	OPSCC	Oropharynx	Cytobrush (control); OPSCC tissue biopsy	Species specific microbial construct	Designed construct for *P. melaninogenica*, *Fusobacterium naviforme*, *Streptococcus anginosus*	52
Takahashi et al. (2019)^75^	Japan	OSCC	Oral cavity	Stimulated saliva	Total bacterial spectrum and abundance ratio	PCR primers targeting 16S rRNA gene's V1–V3 regions	140
Chen et al. (2020)^87^	Hong Kong	HNSCC	Oral cavity, oropharynx, larynx	Oral rinse samples, tissue biopsy	Total bacterial spectrum and abundance ratio	PCR primers targeting 16S rRNA gene's V1–V3 regions (341F/806R)	272
Debelius et al. (2020)^82^	China	NPC	Nasopharynx	Saliva samples	Total bacterial spectrum and abundance ratio	PCR primers targeting 16S rRNA gene's V1–V3 regions (341F/805R)	994
Song et al. (2020)^116^	China	OSCC	Oral cavity	Unstimulated saliva	Not specified	Conductive polymer ionization mass spectrometry	373
Zhang et al. (2020)^76^	China	OSCC	Buccal mucosa	Oral swabs from tumor and normal tissues	Total bacterial spectrum and abundance ratio	PCR primers targeting 16S rRNA gene's V1–V3 regions (338F/806R)	100
Zhou et al. (2020)^73^	China	OSCC	Oral cavity	Tissue biopsy samples	Total bacterial spectrum and abundance ratio	PCR primers targeting 16S rRNA gene's V3–V4 regions (341F/806R)	48
Zou et al. (2020)^117^	China	HNSCC	Larynx, hypopharynx	Unstimulated saliva	Total bacterial spectrum and abundance ratio	PCR primers targeting 16S rRNA gene's V1–V3 regions (341F/806R)	120
Banavar et al. (2021)^118^	USA	OC, OPMD	Oral cavity	Saliva samples	Total bacterial spectrum and abundance ratio	PCR primers targeting 16S rRNA gene's V3–V4 regions	362
Chen, Chen et al. (2021)^119^	Taiwan	OSCC	Oral cavity	Unstimulated saliva	Total bacterial spectrum and abundance ratio	PCR primers targeting 16S rRNA gene's V3–V4 regions	52
Eun et al. (2021)^106^	Republic of Korea	OSCC	Oral cavity	Saliva samples	Total bacterial spectrum and abundance ratio	PCR primers targeting 16S rRNA gene's V3–V4 regions (341F/805R)	54
Gopinath et al. (2021)^72^	India	OSCC, OPMD	Floor of the mouth, buccal mucosa, tongue, gingiva	Unstimulated saliva	Total bacterial spectrum and abundance ratio	PCR primers targeting 16S rRNA gene's V1–V3 regions (319F/806R)	74
Granato et al. (2021)^80^	Brazil	OSCC	Oral cavity	Unstimulated saliva	Total bacterial spectrum and abundance ratio	PCR primers targeting V3–V4 regions of 16S rRNA gene (515F/806R)	24
Monedeiro et al. (2021)^120^	Poland	OSCC	Oral cavity	Unstimulated saliva	Not specified	Gas chromatography mass spectrometry	30
Neuzillet et al. (2021)^88^	France	OSCC	Oral cavity	Tissue biopsy samples	Species‐specific microbial construct	Targeted primer for *F. nucleatum* detection using LPS mouse monoclonal antibody (Clone C8)	212
Su et al. (2021)^38^	Taiwan	OSCC	Buccal mucosa	Swab samples	Total bacterial spectrum and abundance ratio	PCR primers targeting 16S rRNA gene's V1–V3 regions (515F/806R)	232
Torralba et al. (2021)^78^	Poland	OSCC	Tonsil, throat, floor of the mouth, tongue	Saliva samples, tissue biopsy	Total bacterial spectrum and abundance ratio	Adaptor‐ligated 16S primers targeting V4 region	59
Zhou et al. (2021)^121^	China	OSCC	Oral cavity	Oral rinse samples, tissue biopsy	Total bacterial spectrum and abundance ratio	PCR primers targeting 16S rRNA gene's V3–V4 regions (341F/806R)	68
Braz et al. (2022)^122^	Brazil	OSCC	Oral cavity	Unstimulated saliva	Total bacterial spectrum and abundance ratio	Measured with electronic tongue microfluid	27
Chen, Wu et al. (2022)^10^	Taiwan	OSCC	Oral cavity	Unstimulated saliva	Total bacterial spectrum and abundance ratio	PCR primers targeting 16S rRNA gene's V3–V4 regions	54
Saxena et al. (2022)^53^	USA	OSCC	Oral cavity	Swab samples	Total bacterial spectrum and abundance ratio	PCR primers targeting 16S rRNA gene's V3–V4 regions (341F/534R)	66
Banavar et al. (2023)^123^	USA	OSCC, OPSCC	Oral cavity, oropharyngeal	Unstimulated saliva	Total bacterial spectrum and abundance ratio	RNA extraction with silica beads, DNase treatment, Illumina sequencing	1175
Meng et al. (2023)^124^	China	ESCC	–	Unstimulated saliva	Bacteroides, Firmicutes, Proteobacteria, Fusobacteria, Actinobacteria	PCR primers targeting 16S rRNA gene's V3–V4 regions	8000
Nouri et al. (2023)^40^	Republic of Korea	OSCC, HNC, PC, GC	Oral cavity, Head and neck region, pancreas, stomach	Unstimulated saliva, Blood	Total bacterial spectrum and abundance ratio	PCR with primers for SCFA, FFAR2, IL‐6, STAT3, TNFAIP8 analysis	1054
He et al. (2024)^125^	China	OSCC	Oral cavity	Unstimulated saliva	Total bacterial spectrum and abundance ratio	PCR primers targeting 16S rRNA gene's V3–V4 regions (341F/806R)	259
Unlu et al. (2024)^90^	Turkey	OSCC	Oral cavity	Unstimulated saliva, Tissue biopsy	Total bacterial spectrum and abundance ratio	PCR primers targeting 16S rRNA gene's V3–V4 regions	22
Praveen et al. (2024)^91^	Republic of Korea	OC	Oral cavity	Unstimulated saliva	Total bacterial spectrum and abundance ratio	PCR primers targeting 16S rRNA gene's V3–V4 regions (515F/806R)	1022
Cai et al. (2024)^92^	China	OSCC	Oral cavity	Tissue biopsy	PCR with primers for HPV infection	PCR primers targeting 16S rRNA gene's V3–V4 regions (341F/806R)	98

Abbreviations: ESCC, esophageal squamous cell carcinoma; HNSCC, head and neck squamous cell carcinoma; HPV, human papillomavirus; OC, oral cancer; OPSCC, oropharyngeal squamous cell carcinoma; OSCC, oral squamous cell carcinoma.

Comparative analyses of microbial composition in OSCC and premalignant lesions have provided evidence for distinct microbial signatures. For example, a significant depletion in *Actinobacteria* and *Cyanobacteria* from OSCC tissues versus adjacent noncancerous tissue has been widely reported to date.^73^ This pattern is mirrored by findings of decreased *Streptococcus*, *Haemophilus*, *Porphyromonas*, and *Actinomyces* and increased *Fusobacterium* in OSCC samples.^74^ These observations are in line with the results of other studies^75^, ^76^ that report higher levels of *Peptostreptococcus*, *Capnocytophaga*, *Alloprevotella*, and *Fusobacterium* and lower levels of *Rothia*, *Streptococcus*, and *Veillonella* in OSCC patients. In another study, Radiac et al.^77^ utilized 16S sequencing of oral swabs and tissue samples, revealed distinct microbial shifts, including changes in *Proteobacteria* and *Firmicutes* abundance and specific species like *Enterococcus cecorum*. These species progress from healthy oral mucosa to dysplasia and OSCC, suggesting early detection potential and the role of periodontal pathogens in cancer progression. These consistent trends may indicate the possible modulatory role of certain bacterial species in OSCC development and point to *Fusobacterium* and *Peptostreptococcus* as probably the most active players in OSCC development.^75^

The composition of the microbiome in OSCC patients has been studied, and some bacterial genera have been identified that could be used as biomarkers for the detection of the disease.^70^ More precisely, Torralba et al.^78^ detected extreme increases in *Prevotella* in the saliva samples of OSCC patients. Also, Hashimoto et al.^79^ found higher levels of *Bacteroidetes* and *Solobacterium* in OSCC tissue than in leukoplakia. Furthermore, in a longitudinal study to analyze microbiota before and after surgery, Granato et al.^80^ documented that *Abiotrophia*, *Acinetobacter*, *Peptostreptococcus*, and *Staphylococcus* remained at increased levels in OSCC patients compared with healthy controls. Moreover, a community analysis revealed that both primary and metastatic HNSCC tissues exhibit consistent microbial alterations in the tumor microenvironment compared to normal tissues, notably an increase in Fusobacteria and a decrease in Firmicutes and Actinobacteria, with an additional increase in Proteobacteria in metastatic samples.^81^ These findings strongly suggest that oral microbiota profiling can be useful in the development of novel, noninvasive diagnostic modalities for OSCC. Apart from the simple presence of bacteria, the impact of microbial communities on tumor progression also includes functional changes in the microbiome. Notably, Su et al.^38^ reported significant changes in bacterial diversity and function in patients with OSCC, including enrichment in *Fusobacterium* species and depletion in *Streptococcus* species. These changes in microbial activity were postulated to promote tumorigenesis by modulating the inflammatory and metabolic signaling cascades. Accordingly, the authors pointed out several bacterial genera, such as *Streptococcus*, *Fusobacterium*, *Peptostreptococcus*, and *Campylobacter*, and specific species, like *Streptococcus pneumoniae* and *F. nucleatum*, as potential diagnostic indicators. These findings support the idea that microbial dysbiosis causes both compositional imbalance and functional alterations that may favor tumor growth and point out that there is a strong correlation between microbial function and OSCC pathogenesis.

The relation of the oral microbiome with nonmalignant head and neck cancers has been explored, but findings have been inconsistent.^79^ Debelius et al.^82^ explored nasopharyngeal cancer based on 16S rRNA sequencing and demonstrated that the microbes were less diversified in the nasopharyngeal cancer patients than in healthy controls. Similarly, Hayes et al.^83^ explored the microbiome of patients with pharyngeal, laryngeal, and oral cancer and observed that *Corynebacterium*, *Kingella*, and *Neisseria* had an inverse association with the risk of laryngeal cancer. They did not observe specific bacterial genera associated with either the oral cavity or pharyngeal cancer types.^83^ These findings, therefore, suggest that the oral microbiome is likely to influence specific subtypes of HNSCC, but the relationship with oral cancer is very complex and requires further exploration.

A growing body of evidence points to the major contribution of oral microbiota in the development of HNSCC, including OSCC.^28^, ^74^, ^84^ Inflammation is a fundamental physiological process implicated in numerous pathologies, including infectious diseases like periodontitis and various cancers, and systemic inflammation indices derived from blood cell counts, such as neutrophil to lymphocyte ratio, platelet to lymphocyte ratio, delta neutrophil index, and systemic immune inflammation index, serve as crucial biomarkers for quantifying inflammation, monitoring its severity in relation to periodontitis and cancer progression, and assessing treatment responses.^85^ *Fusobacteria* have been shown to be increased in the progression of OSCC, whereas *Actinobacteria* and *Bacteroidetes* are decreased.^74^ On the other hand, high levels of *Bacteroidetes* are related to mutational clusters and thus may indicate more advanced stages of cancer with larger tumor sizes. Further analysis indicated that higher levels of *Fusobacterium*,^86^ *Rothia*,^75^ and *Actinomyces* ^87^ were correlated with lower T‐stages; however, low levels of *Parvimonas* were found in early‐stage tumors.^86^ On the other hand, *Peptostreptococcus* abundance was positively correlated with advanced stages of disease,^87^ whereas *Veillonella* is found to play a protective role as it is inversely related to tumor size, number of lesions, and clinical stage of HNSCC patients.^80^ Additional investigation by Neuzillet et al.^88^ showed that *F. nucleatum* is related to lower T‐stages, minor toll‐like receptor 4 expression, and lesser M2 macrophage recruitment, thus pointing possibly to better clinical outcomes. Su et al.^38^ further reported that oral dysbiosis impairs the production of anticancer compounds, including siderophores and monoterpenoids, representing a complex relationship between microbial function and the progression of OSCC.

Role of oral microbial dysbiosis in the progression of oral cancer has been further investigated by recent studies. A recent review by Zhou et al.^89^ demonstrated that periodontal pathogens, including *P. gingivalis*, *F. nucleatum*, and *T. denticola*, are implicated in cancer development by directly and indirectly influencing chronic inflammation, immune responses, cell proliferation, and anti‐apoptotic activity, suggesting that further understanding their pathogenic mechanisms could lead to novel diagnostic, preventive, and therapeutic strategies. Unlu et al.^90^ studied the comparative analysis of oral microbiota between patients of oral cavity cancers and healthy controls, yielding significant microbial shifts. The results included decreases in *Proteobacteria*, *Aggregatibacter*, *Haemophilus*, and *Neisseria* and increases of *Firmicutes*, *Bacteroidetes*, *Actinobacteria*, *Lactobacillus*, *Gemella*, and *Fusobacteria*. In species‐level results, a decrease in *Campylobacter durum*, *Lactobacillus umeaens*, *Neisseria subflava*, *Aggregatibacter massiliensis*, and *Veillonella dispar* was shown, while the species *Gemella haemolysans* was significantly increased. These findings provide deeper insight into the microbial landscape associated with oral cancer progression. Further, the study revealed very striking increases in major periodontopathogens, including a 6.5‐fold and 2.8‐fold increase in *P. gingivalis* and *F. nucleatum*, respectively. These findings emphasize their pivotal role in driving pro‐inflammatory processes and tumorigenesis.

Moreover, the study by Praveen et al.^91^ supports the hypothesis on the interaction of oral microbiome, fatty acid metabolism, and prognosis in OSCC. With the help of ML modeling and 16S rRNA sequencing, it was possible to identify higher levels of *Streptococcus* and *Parvimonas* in individuals with oral cancer, while *Corynebacterium* and *Prevotella* showed an opposite trend. Incremented levels of the pro‐inflammatory cytokine, interleukin‐6 (IL‐6), and tumor necrosis factor‐alpha (TNF‐α) were positively related to the level of fatty acid beta‐oxidation enzymes, mainly represented by Carnitine palmitoyltransferase 1A (CPT1A). On the other hand, healthy subjects had the highest concentration of short‐chain fatty acids (SCFAs) related to increased concentrations of CD4+ T‐helper cells. Further survival analysis indicated that the presence of *Streptococcus* and *Parvimonas* is associated with a low DFS as well as overall survival, while the presence of *Prevotella* and *Corynebacterium* was correlated with positive effects. These results underline not only the necessity of a holistic approach to be developed within further research but also indicate that microbiota community‐altered metabolic and immunological pathways modify oral cancer progression.

In an effort to investigate the association between oral microbiome and OSCC, Cai et al.^92^ performed an integrative analysis on mucosal bacterial communities, host transcriptomes, and DNA CpG methylation profiles in OSCC patients. They were able to identify seven bacterial species that are significantly enriched in the tumor microenvironment, including *F. nucleatum*, *T. medium*, *Peptostreptococcus stomatis*, *Gemella morbillorum*, *Catonella morbi*, *Peptoanaerobacter yurli*, and *Peptococcus simiae*. In particular, these bacteria showed 254 positive correlations with 206 host genes that were upregulated and involved in cell adhesion, migration, and proliferation regulatory pathways. Further DNA methylation pattern analysis revealed at least 20 host genes with inverted CpG methylation profiles within their corresponding promoter regions, suggesting one possible means by which pathogenic bacteria may manipulate host gene expression via epigenetic modifications. Tezcan et al.^93^ explored the intricate role of chronic inflammation in cancer pathogenesis and treatment challenges. They highlighted recent strategies that leverage specialized pro‐resolving mediators to actively resolve inflammation, modulate the tumor microenvironment, and restore tissue homeostasis, offering a promising alternative to traditional anti‐inflammatory therapies.

The findings of these studies present clear evidence of various complexities between oral microbiota dysbiosis and host metabolism, as well as immune and genetic aspects in cancer initiation and development. A thorough understanding of these intricate interactions not only provides invaluable insights into the pathogenetic mechanisms underlying OSCC development but also emphasizes the potential use of these microbial and molecular signatures for both diagnostic and therapeutic purposes.

### 3.3 Oral microbiota, inflammation, and DNA damage response in oral cancer

Dysbiosis of the oral microbiome, characterized by changes in microbial community composition, structure, and function, has been identified as a critical factor in the development and progression of oral cancer through multiple mechanisms, including increased microbial growth, modulation of host signaling pathways, and production of various bacterial toxins and virulence factors that induce and perpetuate chronic inflammation and carcinogenic DNA damage.^94^ This microbial dysbiosis disrupts the delicate balance between the host and resident microbiome, mostly compromising the integrity of the mucosa barrier through the activation of pattern recognition receptors, specifically Toll‐like receptors along with their downstream signaling cascades. These include but are not limited to nuclear factor kappa‐light‐chain enhancer of activated B cells (NF‐κB) and MAPK pathways.^95^ Disruption of the mucosal barrier provides an avenue through which bacteria and bacterial products may pass into the tissues and further contribute to inflammation of a cancer‐promoting nature. The following bacterial species have been implicated in oral carcinogenesis: *F. nucleatum* has been strongly implicated in the development and progression of oral cancer. Experimental in vitro paradigms also validated the capability of *F. nucleatum* to induce cellular invasion through regulation of the E‐cadherin/β‐catenin signaling cascade and the TNF‐α/NF‐κB pathway and by enhancing remodeling of the extracellular matrix via upregulation of the SNAI2 gene, a pivotal transcription factor involved in epithelial–mesenchymal transition.^92^ *Fusobacterium nucleatum* infection induces NF‐κB signaling, one of the major transcription factors in inflammatory responses, which further causes increased production of pro‐inflammatory cytokines such as IL‐6 and TNF‐α and activation of NLRP3 inflammasome, a multiprotein complex involved in innate immunity and inflammation.^96^ This inflammasome activation results in caspase‐1 mediated IL‐1β production, a potent pro‐inflammatory cytokine that further amplifies the inflammatory response and creates a pro‐tumorigenic microenvironment. Interestingly, *F. nucleatum* can activate NLRP3 inflammasome in gingival epithelial cells in an ATP‐independent manner, which is different from most other pathogens requiring extracellular ATP for inflammasome activation. This pathogen also releases damage‐associated molecular patterns (DAMPs) such as high mobility group box 1 protein (HMGB1), a nuclear protein that can be released into the extracellular space upon cellular damage or stress and act as a potent pro‐inflammatory mediator that further exacerbates inflammation and creates a tumor‐promoting microenvironment.^96^ It has been demonstrated that infection with *F. nucleatum* causes DNA double‐strand breaks in tongue squamous cell carcinoma, as evidenced by increased γ‐H2AX, and impairs mechanisms of DNA repair through the downregulation of Ku70, a key protein involved in nonhomologous end joining, and wild‐type p53, a tumor suppressor protein implicated in DNA damage response and cell cycle control. These disruptions in DNA integrity and repair capacity lead to genomic instability, a hallmark of cancer that accelerates cancer progression and promotes acquisition of further genetic mutations.^23^, ^97^

Apart from the direct DNA damage produced by some, other microorganisms contribute to carcinogenesis by producing carcinogenic metabolites and inducing chronic inflammation. *Porphyromonas gingivalis* is a keystone periodontitis pathogen that produces several metabolites with carcinogenic potential, including oxygen radicals, such as superoxide anion, hydroxyl radical, butyrate, and acetaldehyde. Butyrate has been demonstrated to induce programmed cell death in T and B lymphocytes, hence compromising host immune surveillance and permitting the tumor cells to evade the immune system. Oxygen radicals cause direct DNA damage by mechanisms including double‐strand breaks, single‐strand breaks, and base modifications. Acetaldehyde enhances DNA damage and increases the proliferation of epithelial cells, thus leading to oral lesions and possible malignant transformation.^98^ The *Prevotella intermedia* produces some volatile sulfur compounds; for example, hydrogen sulfide (H_2_S) and methyl mercaptan (CH_3_SH), which inflict oxidative stress on periodontal pockets, leading directly to DNA damage. Hydrogen sulfide inhibits superoxide dismutase activity and the antioxidant potency of superoxide dismutase to convert superoxide radicals to less harmful ones; thus, it generates oxidative stress due to ROS accumulation. Methyl mercaptan has been shown to facilitate tumor invasion into the surrounding tissue by degrading type IV collagen, the major component of the basement membrane, allowing the migration and invasion of tumor cells into the surrounding tissue.^23^, ^99^ Other mechanisms involved include chronic inflammation, which is a hallmark of periodontitis as well as other oral inflammatory disorders, which also play a significant role in oral carcinogenesis. Chronic inflammation allows the acquisition of genetic mutations, impairs mechanisms of DNA repair, and promotes a tumor‐supportive microenvironment with growth factors, cytokines, and chemokines.^99^ Chronic inflammation caused by periodontitis can impact various regions of the body, such as the oral cavity, head and neck area, lungs, esophagus, and stomach, potentially initiating cancer in distant organs via systemic blood circulation. This occurs through mechanisms such as the stimulation of cell proliferation and enhancement of mitogenic activities.^100^ These processes are driven by the activation of signaling pathways like MAPK/ERK, suppression of apoptosis, induction of DNA damage in rapidly dividing cells, and the release of pro‐inflammatory mediators, including cytokines like IL‐6, IL‐8, IL‐1β, TNF‐α, and other chemokines.^101^ The reactive oxygen and nitrogen species produced by inflammatory cells like neutrophils and macrophages directly damage DNA through various mechanisms, including strand breaks, base modifications, DNA adduct formation, and oxidative damage to DNA bases.^102^ Over time, these genotoxic effects lead to genetic alterations, such as mutations and chromosomal rearrangements, which, in turn, favor malignant transformation and genomic instability. Besides the DNA‐damaging ability, inflammation promotes the inhibition of apoptosis and stimulates cell proliferation. This further accelerates cancer development and tumor growth.^103^ All these mechanisms give an idea of the important and complex role that oral microbiota may play in oral cancer development. By the induction of microbial dysbiosis, chronic inflammation, and direct and indirect DNA damage, the microbiome contributes to genomic instability, tumor development, and disease progression. These call for targeted interventions aimed at the restoration of microbial homeostasis, mitigation of inflammation, and, finally, reduction of cancer risk.

### 3.4 Oral microbiota as a diagnostic or prognostic tool for oral cancer

A large body of research has focused on identifying the prognostic significance of oral microbiota composition in OSCC.^80^, ^87^, ^88^, ^104^ For example, Granato et al.^80^ found that several different bacterial species, for example, *Stenotrophomonas*, *Staphylococcus*, *Centipeda*, *Selenomonas*, *Alloscordovia*, and *Acinetobacter*, are inversely correlated with survival rates in OSCC patients. Moreover, it is observed that *Veillonella* is also inversely correlated with tumor size, thus suggesting a more favorable prognosis. In line with these findings, Neuzillet et al.^88^ reported that OSCC patients with *F. nucleatum*‐positive tumors showed significantly longer relapse‐free and metastasis‐free survival compared to those without *F. nucleatum*. This bacterium was further associated with improved three‐year disease‐specific and disease‐free survival of the patients, particularly nonsmokers and those suffering from early‐stage tumors with low recurrence rates. Reduced abundance of bacterial genera *Neisseria*, *Haemophilus*, and *Rothia* was found to decrease cancer‐specific survival in cases of HNSCC.^87^ Moreover, Robayo et al.^104^ found a nonstatistically significant trend for poorer survival in HPV‐positive OSCC patients who were co‐infected with *Streptococcus anginosus*.

Praveen et al.^91^ further enriched these findings by showing that increased abundance of *Streptococcus* and *Parvimonas* in individuals with oral cancer was associated with increased levels of inflammatory cytokines, including IL‐6 and TNF‐α, known to enhance tumor progression. Moreover, their study indicated that higher levels of *Streptococcus* and *Parvimonas* were significantly correlated with poorer disease‐free survival and overall survival. This finding suggests a possible mechanistic association between dysbiosis of the microbiome, inflammation, and poor clinical outcomes. On the contrary, reduced levels of *Prevotella* and *Corynebacterium* are associated with improved probabilities of survival, which points out the two‐edged and complex role of the microbiome in modulating oral cancer prognosis and patient outcome. Notably, the genus *Capnocytophaga* was found to be significantly enriched in OSCC patients with disease recurrence after surgical tumor resection, with relative abundance levels more than five‐fold higher compared to those in healthy control subjects. This indicates its value as a potential biomarker for predicting cancer recurrence.^105^ Similarly, Eun et al.^106^ conducted a parallel study to explore salivary microbiome composition as a noninvasive biomarker predictive of lymph node (LN) metastasis in OSCC, and their random forest model showed a high level of accuracy for predicting metastasis. Thus, these findings support the high potential of microbiome‐based biomarkers in oral oncology for further stratification of a patient's risk toward metastasis and disease progression to more tailored treatments.

A summary of all the studies on the prognostic role of oral microbiome in oral cancer is given in Table 2. The collective findings from many studies show that oral microbiome changes in both precancerous lesions and established OSCC, with specific bacterial genera consistently showing up across different studies, suggesting an etiologic role. Microbial dysbiosis, where certain bacterial taxa dominate and others decline, seems to create a microenvironment that favors tumorigenesis by modulating inflammatory and metabolic signaling pathways.^70^ While the exact molecular mechanisms need to be further investigated, these findings strongly suggest the microbiome plays a big role in the diagnosis and progression of oral cancer. Identifying and functionally characterizing microbial biomarkers in the oral cancer microenvironment offers opportunities for the development of microbiome‐targeted therapeutic and preventive strategies. Large‐scale longitudinal studies and mechanistic studies are needed to validate the associations, establish causality, and translate these findings into clinical applications.

**TABLE 2:** Summary of key studies investigating the prognostic impact of oral microbiota in HNSCC and OSCC.

Author (year)	Cancer type	Microbial sample types	Analysis target	Microbial findings	Clinical correlation	Prognostic / diagnostic insights	Statistical method
Warnke‐Sommer et al. (2017)^107^	OC	Swab samples	Bacterial and functional gene distribution using SVM models	↑ Fusobacterium, Prevotella; ↓ Streptococcus, Rothia, Haemophilus	Altered microbiome linked to carcinogenesis	Potential biomarker for early detection of OSCC	PCA, Welch's *t*‐test, SVM with ROC AUC evaluation
Ganly et al. (2019)^105^	OSCC	Oral rinse samples	Relative abundance and Recurrence	↑ *Capnocytophaga*	Recurrent tumors	Unfavorable: linked to recurrence	Kruskal–Wallis, Mann–Whitney
Robayo et al. (2019)^104^	OSCC	Cytobrush and tissue biopsy	HPV+ patients and HPV+/*Streptococcus anginosus* patients	*S. anginosus* (not statistically significant)	HPV+ with shorter survival	Potentially unfavorable for HPV+ patients	Kaplan–Meier, Log‐rank
Chen et al. (2020)^87^	HNSCC	Oral rinse and tissue biopsy	Relative abundance and clinical prognostic factor	↑ *Fusobacterium nucleatum*	Better DSS and DFS	Favorable: lower T stage; potential biomarker	Uni‐ and multivariate
Granato et al. (2021)^80^	OSCC	Unstimulated saliva	Relative abundance and clinical prognostic factor	↑ Stenotrophomonas, Staphylococcus, Selenomonas, Centipeda, Alloscardovia, Acinetobacter	Correlated with tumor size/stage	Unfavorable: Predictive of lower OS	Pearson, chi‐squared
Neuzillet et al. (2021)^88^	OSCC	Tissue biopsy	Intratumoral *F. nucleatum* and clinical prognostic factor	*F. nucleatum* (merged)	Lower recurrence in nonsmokers	Favorable: improved OS, RFS, and MFS	Kaplan–Meier, Chi‐squared
Banavar et al. (2021)^118^	OC, OPMD	Saliva samples	Metatranscriptomic features in saliva	↑ Porphyromonas and Fusobacterium	Early detection of OC	Potentially favorable: linked with cancer risk	Machine learning classifier evaluation
Eun et al. (2021)^106^	OSCC	Saliva samples	Lymph node metastasis and salivary microbiome	↑ Prevotella, Bifidobacterium	Lymph node metastasis	Unfavorable: correlated with metastasis risk	Random forest classifier, diversity analysis
Saxena et al. (2022)^53^	OSCC	Unstimulated saliva	Impact of smokeless tobacco on oral microbiome	↑ Prevotella, *Capnocytophaga*, Fusobacterium; ↓ Streptococcus in OSCC and tobacco users	Inflammationprone microbiome, higher cancer risk	Diagnostic potential in smokeless tobacco users and OSCC risk assessment	α‐diversity (Shannon, Simpson, Chao1); β‐diversity (UniFrac, Bray–Curtis); genus identification with LEfSe and Boruta
Nouri et al. (2023)^40^	OSCC, HNC, PC, GC	Unstimulated saliva, Blood	Bacterial impact on inflammation and immune response	↓ Streptococcus, Abiotrophia, Leuconostoc, Prevotella; ↑ Haemophilus, Neisseria	Increased risk of cancer due to inflammation	Potential for microbiome‐targeted cancer prevention	Machine learning (GLM, RF, GBM), diversity metrics, logistic regression, LEfSe, Spearman's correlations in R
He et al. (2024)^125^	OSCC	Unstimulated saliva	Microbial dysbiosis index for OSCC prediction	↑ Streptococcus, Capnocytophaga, Gemella in OSCC; ↓ Lachnospiraceae, Veillonella	Diagnostic potential in OSCC risk	Noninvasive, high‐accuracy prediction with Random Forest classifier	Random Forest classifier, AUC evaluation
Praveen et al. (2024)^91^	OSCC	Unstimulated saliva	Fatty acid metabolism, microbial composition	↑ Streptococcus and Parvimonas; ↓ Corynebacterium and Prevotella	Elevated inflammatory markers (IL‐6, TNF‐α) and altered microbiome linked to progression	Unfavorable: ↑ Streptococcus and Parvimonas correlated with poorer DFS/OS; Potential biomarker for diagnosis and prognosis	Light Gradient Boosting Machine (LightGBM); *t*‐tests, chi‐squared tests, OTUs, Chao index, PCoA (weighted/unweighted UniFrac), Cox regression (uni−/multivariate), Wilcoxon rank sum, logistic regression, LEfSe

Abbreviations: AUC, area under the curve; HNC, head and neck cancer; HNCC, head and neck cell carcinoma; HNSCC, head and neck squamous cell carcinoma; HPV, human papillomavirus; OC, oral cancer; OPSCC, oropharyngeal squamous cell carcinoma; OSCC, oral squamous cell carcinoma; PCA, principal component analysis; ROC, receiver operating characteristic.

## 4 ORAL MICROBIOME SAMPLE COLLECTION AND CURATION

### 4.1 Oral microbiome sample collection

The literature shows that researchers have employed different sampling methods to study the relationship between the oral microbiome and oral cancer. Each of these methods has its advantages and disadvantages in capturing the whole microbiome.^126^, ^127^ Generally, the process of molecular characterization of the microbiome begins with acquiring nucleic acids from various sample types. Subsequently, the composition, structure, functional potential, and spatial distribution of microbial communities in the oral cavity and their correlation with carcinogenesis and tumor progression are extracted.^128^ Liquid biopsy is a transformative, noninvasive diagnostic tool that analyzes genetic biomarkers like ctDNA, CTCs, and exosomes in bodily fluids, for example, blood, saliva. It is used to predict susceptibility, facilitate early detection, and enable real‐time monitoring of cancer progression and treatment response, especially for late‐stage diagnoses like OSCC, with periodontists and hygienists playing a crucial role in its future screening implementation.^129^ However, these sampling methods are intrinsically prone to several limitations and biases, which can seriously impact the accuracy, reproducibility, generalizability, and interpretability of resultant findings.

Unstimulated whole‐mouth saliva, collected without any exogenous stimulation of salivary flow, gives a static or resting state representation of the saliva's natural biochemical composition (electrolytes, proteins, enzymes, other biomolecules) and resident microbial communities, a mixed population from various oral surfaces.^130^, ^131^ This method gives valuable baseline data of the resting oral microbiome which can help in detecting microbial shifts and dysbiotic states associated with oral carcinogenesis. Although this gives a noninvasive and easy to get baseline, interindividual variations in unstimulated salivary flow rates due to factors, like age, sex, circadian rhythms, medications, systemic health conditions, and environmental factors like dietary intake, hydration status, oral hygiene practices, exposure to environmental toxins, etc., on salivary constituents can be confounding variables that can introduce noise and variability in the data and can mask the biological signals.^132^ Furthermore, it is important to acknowledge that saliva, as a composite fluid collected from the entire oral cavity, may not accurately reflect the microbial populations residing within specialized and distinct microenvironments of the oral cavity, such as the gingival sulcus (the crevice between the tooth and the gingiva, a key site for periodontal disease development) or the various mucosal surfaces lining the oral cavity (including the buccal mucosa, tongue, and palate), which may harbor distinct microbial communities relevant to oral carcinogenesis. Stimulated saliva samples obtained by inducing salivary flow through exogenous stimuli such as mastication (chewing) or topical application of citric acid or other gustatory stimuli provide a more dynamic and potentially comprehensive microbial profile that may capture a wider range of microbial taxa including transient or low abundance species associated with oncogenic processes and tumor development.^130^ This technique has the advantage of higher microbial yield and may capture transient microbial populations but also introduces variability from differences in stimulation protocols (type, duration, intensity of stimulation and the specific stimulus used) and may preferentially sample microbes from the major salivary glands, for example, parotid, submandibular, and sublingual glands, rather than the epithelial surfaces of the oral cavity and may introduce sampling bias and skew the representation of the true microbial community structure in specific and clinically relevant oral microenvironments.^133^

Beyond saliva, other sampling strategies are used in oral microbiome research. Oral rinse samples are collected by having participants rinse with a standardized solution such as saline or phosphate‐buffered saline and collect cellular material and microorganisms from many oral sites.^134^, ^135^ This method provides a broad representation of the oral microbial community and, therefore, may be beneficial for identifying potential microbial biomarkers associated with cancer‐relevant processes. One must consider methodological issues in this context, as possible dilution of microorganism content during the rinsing procedure may prevent the detection of underrepresented microbes, limiting the sensitivity of analyses downstream. Moreover, variations in rinse time, rinse volume, and different compositions of solution can all influence recovery rates and create sources of variability between studies, leading to comparison challenges across studies. Additionally, variations in rinse time, volume of rinse solution, and the composition of the solution itself can impact microbial recovery rates and introduce inter‐study variability that can make it difficult to compare across studies. In contrast to oral rinse samples, tissue biopsies obtained by direct excision of tissue from suspected malignant lesions or adjacent healthy tissue provide a highly localized and targeted view of the microbial community at the tumor site.^136^, ^137^ This allows for the study of microbial effects on tumor growth, invasion, and metastasis. However, the invasiveness of biopsy procedures limits their clinical applicability, especially in vulnerable patient populations with comorbidities or advanced disease stages. Moreover, the risk of contamination from adjacent tissues during the excision process or from surgical instrumentation is a potential confounder that can compromise the sample. The limited amount of tissue material obtained from biopsy also restricts the scope of downstream analysis and poses a challenge for comprehensive analysis of both microbial and host‐derived molecular components.

The collection of samples by oral microbiome sampling is highly selective; an oral swab collects microbial material from discrete anatomical regions of the oral cavity, including sites of healthy and neoplastic tissue.^137^ This procedure allows one to make direct intraindividual microbial profile comparisons; it further allows estimates of site‐specific microbial variation for any given individual. From a methodological point of view, some issues should be put into consideration while using oral swabs for microbiome sampling. By their design, passive methods may miss much of the microbial diversity truly present in the oral cavity and grossly underestimate the microorganisms existing either in the deeper mucosal layers or even in subgingival plaque present in this concealed environment. These minor variations in pressure while swabbing, and slightly different swabbing techniques utilized by different operators, may easily be magnified into major variations in the amount and/or composition of microbial material collected from one sample to another, impacting reproducibility and comparability in downstream analysis. Other biological materials such as tissue samples give more adjunct material toward microbiome studies outside direct oral sampling, owing to their interaction with systemic factors such as inflammatory markers and cytokines circulating in relation to localized shifts in microbial populations, especially in cases of oral cancer.^136^ This systemic view could provide a better insight into which oral dysbiosis impacts the immune response and thereby turns tumorigenesis. Conventional blood samples, however, do not express the oral cavity microbiome completely. Properties of the blood samples are also prone to variability because of possible contamination by skin bacteria upon collection. They should, ideally, be used to work in tandem with oral sampling mechanisms rather than as isolated techniques in the study of oral microbiomes. The noninvasive fresher methods, using cyto‐brushes, may gently scrape the cells from the oral mucosa but preferentially sample from the mucosal surface.^138^ Other less invasive samples such as mouthwash samples capture microorganisms from various oral surfaces, and they compromise microbial yield compared to tissue biopsies and can be contaminated with external microorganisms.

Oral sampling methods vary significantly in their invasiveness and the type of biological information they yield, each presenting distinct advantages, disadvantages, and limitations. Noninvasive approaches like oral rinses and unstimulated saliva are highly patient‐friendly, cost‐effective, and ideal for large‐scale screening or longitudinal studies, offering a general overview of the planktonic and superficial oral microbiome.^139^ While they provide broad insights into the oral cavity's microbial ecosystem, their primary limitation lies in their lack of site specificity and potential for sample dilution. Similarly, oral swabs and cytobrushes offer minimally invasive, targeted collection of superficial cells and microbes from specific mucosal sites or lesions; cytobrushes typically provide a higher cellular yield suitable for more detailed host cell analysis, yet both remain limited to surface‐level information and may not reflect deeper tissue changes.^140^ In contrast, tissue biopsy stands as the gold standard for definitive diagnosis, providing highly localized insights into host tissue architecture and the microbial communities directly embedded within the lesion. Its key advantages include capturing in situ host–microbe interactions and offering high‐quality material for comprehensive genomic and transcriptomic analysis.^136^ However, its significant disadvantages include its invasive nature, requiring local anesthesia and posing risks of pain, bleeding, and infection, thereby limiting its suitability for routine screening or repeated sampling. Ultimately, the selection of an oral sampling method hinges on the specific research question or clinical diagnostic need, with multi‐method approaches often employed to achieve a more holistic understanding.

Microbiome studies often face the challenge of contamination, which can distort microbial profiles and introduce bias, jeopardizing the validity and reliability of results. Contamination primarily arises from external environmental DNA and cross‐contamination during sample processing.^141^ To address this, stringent decontamination protocols, both in the laboratory and during bioinformatics analysis, are essential. For example, blank control samples containing only reagents can detect contaminants introduced during processing, while computational tools can subtract contaminant sequences by comparing experimental samples with controls.^141^, ^142^ Well‐established protocols, such as those used in the Cancer Microbiome Atlas (TCMA) project, have effectively generated high‐quality, reproducible microbiota profiles by removing common contaminants.^141^ However, *in silico* decontamination methods, while useful, cannot replace well‐designed studies with proper controls and meticulous sample handling.^143^ For instance, stringent protocols in breast cancer biopsy studies have improved discrimination between tumor and normal tissue microbiomes. When selecting sample types for cancer microbiome studies, the research question must align with the information each sample type provides. Detailed documentation of the sample processing workflow, including controls, is crucial to address contamination sources. Transparency and methodological rigor are vital for building robust diagnostic models and ensuring the credibility and reproducibility of microbiome data from clinical samples.^143^

### 4.2 Oral microbiome data curation

Detection of taxonomic composition is a critical and essential step in the analysis of microbiome samples, which refers to the identification of a microbial species and their quantification in terms of being present in an environment being sampled.^144^ The taxonomic profile provides extensive as well as a detailed characterization of the structure and diversity of a microbial community and allows the differentiation and quantification of both abundant and rare species within the sample, which provides meaningful insights into the ecological balance of that microbial community.^145^ It has been critical in cancer research as specific microbial community structures, or dysbiosis, have become increasingly implicated in the disease's progression, modulation of the host immune response, and beginning or perpetuation of inflammatory processes that can contribute to tumorigenesis.^146^ A thorough understanding of these complex interrelationships is essential for the identification of potential microbial biomarkers with diagnostic or prognostic value and for the development of targeted therapeutic strategies aimed at modulating the microbiome.

#### 4.2.1 DNA/RNA extraction and sequencing

**FIGURE 3:**
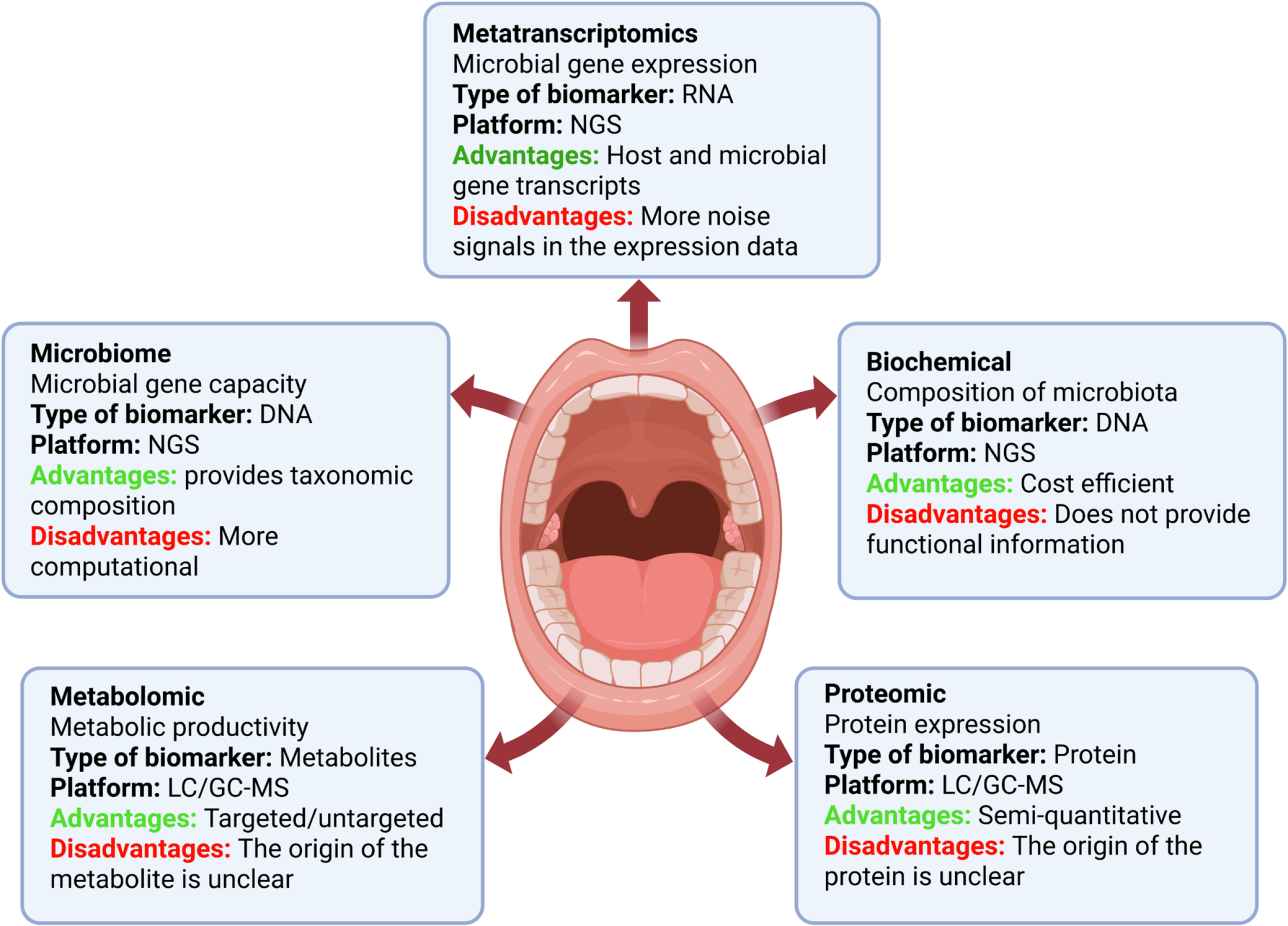
Omics approaches for characterizing the oral microbiome: A set of omics technologies is employed to provide a comprehensive and multifaceted characterization of the oral microbiome. These include *Metagenomics*: This approach analyzes the total DNA extracted from a microbial community, enabling the identification and quantification of the constituent microbial taxa, thus providing a detailed profile of the taxonomic composition of the microbiome. *Metatranscriptomics*: This technique analyzes the total RNA present within a microbial community, revealing the dynamic gene expression profiles of the constituent microorganisms and providing insights into their functional activities under specific conditions. *Metabolomics*: This field focuses on the comprehensive analysis of the complete set of metabolites present within a biological sample, offering valuable insights into the metabolic pathways and biochemical processes occurring within the microbial community. *Proteomics*: This approach involves the large‐scale analysis of the total protein content (proteome) of a microbial community, providing direct information on the functional activities, interactions, and regulatory mechanisms of the constituent microorganisms. *Biochemical Assays*: These assays involve the targeted analysis of specific biochemical components and activities within the microbiota, providing complementary insights into the metabolic activities and physiological state of the microbial community (Created with BioRender.com).

The first crucial step in microbial analyses is a successfully performed quality extraction of DNA or RNA. Figure 3 provides a summary of the approaches applied for oral microbiome analysis. Salivary microbial DNA was extracted by Nouri et al.^40^ using the Fast DNA Spin extraction kit and targeted the V4 region of the 16S rRNA gene for taxonomical profiling. Banavar et al.^118^ carried out metatranscriptomic analysis by mapping RNA reads to over 50 000 microbial genomes and resolving taxa down to the strain level. The 16S rRNA gene sequencing is the principal approach where the functionality of the microbial DNA gene has been analyzed and the taxonomic composition of the microbial community provided. Nouri et al.^40^ related the different bacteria species that are responsible for the progression of oral cancer using 16S rRNA gene analysis and explained associations between microbial dysbiosis and disease outcomes. While taxonomic profiles provide detailed information, they are very computationally demanding to analyze. Another important tool applied is metatranscriptomics, which deals with microbial gene expression as inferred from the RNA transcripts. The approach involves host and microbial gene activities in which genes are actively expressed under certain contextual conditions. The work of Banavar et al.^118^ showed strong associations between active microbial pathways and oral cancer development with greater resolution. In general, a given approach seeks high‐throughput NGS platforms, such as Illumina MiSeq or NovaSeq, because they are known to connect observed microbial activity to distinct metabolic pathways and a plethora of biological processes. What this does share with all transcriptomic and metatranscriptomic studies is the excessive background noise from both active host and microbial‐derived RNA transcripts, which confounds subsequent data analysis and interpretation, often necessitating further development of complex noise‐cleaning methods.

Biochemical analysis analyzes the overall composition of microbiota using DNA as a biomarker. This method provides a cost‐efficient perspective as it can be done using high‐throughput sequencing on NGS platforms. Ganly et al.^105^ used this technique to study microbial diversity and the association with recurrent episodes of oral cancer. However, it does not provide functional information related to the microbiome and might not be able to provide information about dynamic interactions. On the contrary, proteomic analysis examines the expression of proteins to understand microbial activity on the functional level of the organism. It offers insights into semi‐quantitative observations of protein expression through mass‐spectrometry‐based techniques such as liquid chromatography–mass spectrometry. For example, Hashimoto et al.^79^ used this technique to study microbial protein profiles in individuals with oral cancer. However, proteomics cannot always attribute proteins to specific microbes. Finally, metabolomics studies metabolic productivity by exploring metabolites produced by microbial communities. Using platforms like liquid chromatography or gas chromatography–mass spectrometry, it provides both targeted and untargeted metabolite profiles. For instance, Su et al.^38^ used metabolomics to identify specific microbial metabolites associated with inflammation and tumor‐supporting environments in oral cancer patients. A challenge with metabolomics is that the origin of the metabolites is often unclear, so it might be hard to interpret.

#### 4.2.2 Microbiome informatics

OTUs and ASVs. The sequenced microbiome data is then preprocessed and cleaned to produce Operational Taxonomic Units (OTUs) or Amplicon Sequence Variants (ASVs). OTUs offer taxonomic classification at higher taxonomic levels, such as genus or species, and are generated by clustering sequences of similar sequences. On the other hand, ASVs accurately represent the true diversity present in a sample at the single‐nucleotide level. While ASVs have much higher resolution than OTUs, both methods have been widely utilized in the literature. For example, Ganly et al.^105^ employed OTU‐based methods and the QIIME bioinformatic pipeline for the taxonomic classification of generated sequence data, which is quite common in the majority of microbiome studies. In addition, Granato et al.^80^ used USEARCH and UCLUST algorithms for the refinement of taxonomic profiles to achieve higher resolution with high computational efficiency and accuracy for ASV generation.

#### 4.2.3 Taxonomic composition

Taxonomic composition analysis is one of the important steps in microbiome characterization. This essentially encompasses the correct identification and quantification of different microbial taxa present within a biological sample. This process yields a well‐profiled, analytical output representing the microbial community structure that contains a detailed list of the relative abundance of different taxa across each level of taxonomy. Thereby, distinguishing between abundant and rare taxa and giving valuable insight into ecological balance, diversity, and potential functional interactions among microbiota. For example, 16S rRNA gene amplicon sequencing data analysis has identified key bacterial strains and genera implicative in the development and progression of oral cancer.^12^ Further, in another approach, metatranscriptomics analyzed RNA transcripts present in the sample, assessed functional activity, and gene expression of the microbial community and attained strain‐level taxonomic resolution; hence, it was able to establish correlations among different microbial strains and defined contexts of cancer, for example, tumor microenvironment, or stages of disease progression.^118^ Among these, characterization of taxonomic composition gains prime importance in studies related to cancer. These could be modulating host immune response, promoting or inhibiting tumor inflammatory processes, and influencing tumor formation, growth, and metastasis by several mechanisms, including the production of metabolites, modulation of host gene expression, and induction of chronic inflammation.^146^

#### 4.2.4 Diversity metrics

Comparative analysis of microbial communities across different samples or conditions depends on calculating alpha and beta diversity metrics. Alpha diversity is a measure of the richness and evenness within each sample and represents the within‐sample diversity. Examples of widely used alpha diversity measures are the number of observed species, the Chao1 index, the Shannon index, and the Simpson index.^147^ For example, Granato et al.^80^ employed the Shannon index in measuring microbial diversity for OSCC samples. In this way, they provided an overall measure for both richness and evenness within these samples. Richness indices, such as the observed number of species, were also used by Lee et al.^71^ to monitor and measure diversity change between the cancerous and noncancerous groups. This allowed them to pinpoint precisely how the numbers of different taxa change. In contrast to alpha diversity, beta diversity is a comparison between different samples or groups of samples regarding community composition, allowing for the identification of differences that may be linked with various conditions, such as health and disease state, response to treatment, or environmental conditions.^147^ Methods like UniFrac distances and visualization methods, such as principal coordinate analysis, suggest that the microbial communities are clustered or separated based on their phylogenetic and taxonomic composition. Using these beta diversity metrics, UniFrac distances, Ganly et al.^105^ demonstrated that the cancer samples had different microbial community composition compared to the control samples. These obvious differences were further validated with robust statistical tests, including the Adonis test, to show the statistical significance of the difference between groups in regard to the composition of communities.

#### 4.2.5 Statistical analysis and feature selection

Various statistical methods have been used in microbiome studies to identify statistically significant associations between microbial data such as taxonomic abundance, functional profile, and diversity metric with clinical outcomes including status of a disease, treatment response, and survival. For example, Banavar et al.^118^ applied the Mann–Whitney *U* test, which is a nonparametric statistical test used for comparing two independent groups where the data are not normally distributed. This test was performed along with the Benjamini‐Hochberg correction, which is a commonly used method dealing with FDR and the issue of multiple comparisons that arises when numerous statistical tests are done at the same time, to identify the differentially expressed microbial functions between cases and controls. Similarly, in the study by Ganly et al.,^105^ the Kruskal–Wallis test, a nonparametric statistical test for comparing three or more independent groups, was applied followed by post hoc Mann–Whitney *U* tests to perform the pairwise comparisons of specific groups for the identification of significant taxonomic differences in microbial community composition. Then, by applying a powerful supervised ML algorithm based on ensemble learning called random forest models, which build multiple decision trees and aggregate their predictions in order to boost accuracy and robustness, both He et al.^125^ and Ganly et al.^105^ classified samples according to their microbial profiles and determined those bacterial genera or any other microbial taxa that associate most strongly with the cancer progression or any other relevant clinical outcome of interest. Table 2 provides an extended overview and detailed information about the specific statistical methods and ML algorithms applied in each study included in this review.

#### 4.2.6 Functional insights

Functional analysis complements taxonomic profiling to show the metabolic and regulatory roles of the microbiome. Metatranscriptomics shows what genes are being expressed or which metabolic pathways are connected to cancer. Metatranscriptomic analysis showed active microbial functions involved in inflammation and immune responses.^118^ The complete analytical pipeline for microbiome data, from the initial extraction of nucleic acids (DNA or RNA) through sequencing and culminating in functional analysis and interpretation, provides a holistic understanding of microbial communities, their complex interactions, and their associations with oral cancer. Each discrete step within this analytical framework, ranging from the precise determination of taxonomic composition and the calculation of relevant diversity metrics to the application of advanced statistical modeling techniques and the derivation of meaningful functional insights, plays a crucial role in the identification of potential microbial biomarkers with diagnostic or prognostic value and in the elucidation of the microbiome's multifaceted role in the complex processes of cancer development, progression, and metastasis. These findings provide a robust foundation for the development of novel diagnostic tools and rationally designed therapeutic strategies that specifically target the oral microbiome with the ultimate goal of improving patient outcomes, enhancing clinical management, and potentially preventing disease progression.

## 5 AI FOR ORAL MICROBIOME ANALYSIS

The capability of AI in handling complex, high‐dimensional data make it a great tool to extract meaningful information in microbiome studies. Computational algorithms to analyze complex data can help researchers identify disease‐associated microbial patterns more accurately.^148^ Several studies have shown the use of ML in analyzing microbial data for oral cancer detection and prognosis.^40^, ^53^, ^123^, ^149^ Interestingly, AI has demonstrably integrated into clinical settings in recent years in several medical and dental fields, including radiology, pathology, anesthesiology, etc. As evidenced by the US Food and Drug Administration's (FDA) AI/ML‐Enabled Medical Device List, over 1000 AI/ML‐enabled medical devices have been authorized for marketing, underscoring the accelerating adoption and clinical relevance of AI in modern healthcare. This list is expected to grow fast as the FDA actively encourages and authorizes the marketing of innovative, safe, and effective medical devices incorporating AI and ML capabilities within the United States. This growing roster of authorized devices underscores the accelerating adoption and clinical relevance of AI in modern health care.^150^

**FIGURE 4:**
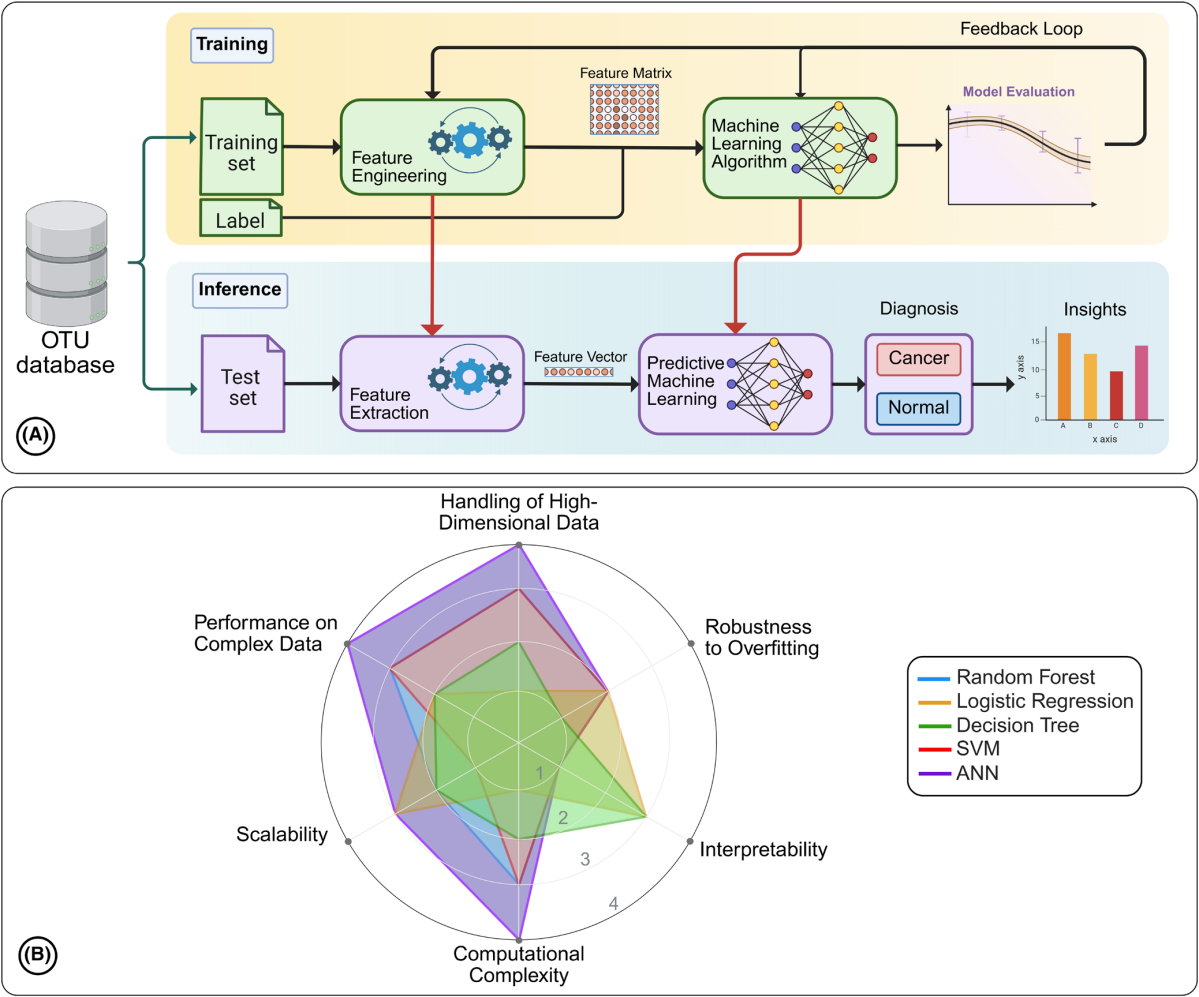
Oral microbiome‐based cancer detection: Machine learning (ML) workflow and algorithm comparison. (A) ML workflow: (1) Data Preprocessing: Raw‐sequencing reads quality control, taxonomic binning or OTU/ASV picking, normalization for sequencing depth, and compositional bias. (2) Feature Engineering/Extraction: Derivation of relevant features, for example, microbial taxonomic abundance, metagenomic functional profiles, α‐ and β‐diversity metrics. (3) Model Training and Evaluation: Train ML models on labeled data sets, using accuracy, precision, recall, F1 score, and AUC for evaluation. (4) Inference: Apply trained models on an independent test data set to classify unseen samples as cancer/normal. (B) Algorithm comparisons: Performance of random forest, logistic regression, decision tree, SVM, and ANN are compared in terms of computational complexity, scalability to large data sets, robustness to overfitting, and interpretability of predictions. (Created with BioRender.com). ANN, artificial neural network; ASV, amplicon sequence variants; AUC, area under the curve; SVM, support vector machine; OTU, operational taxonomic units.

Figure 4 demonstrates a general framework of ML algorithms' role in oral microbiome‐based cancer detection. It broadly consists of three phases, including data preprocessing, training, and inference phases. The training phase includes feature engineering and extraction from labeled training data and training a ML algorithm. The trained model is then used in the inference phase to predict the cancer status of the unseen test data. For example, Banavar et al.^118^ utilized logistic regression with *L*2 regularization to create a classifier based on microbial activity profiles. Other studies such as Granato et al.^80^ and Ganly et al.^105^ used support vector machines or random forest models for feature selection and predictive modeling to understand the relationship between microbial composition and cancer outcomes. Learning‐based models help analyze large volumes of data and enable researchers to build predictive tools for early diagnosis and precision medicine in oncology. The integration of taxonomic data, diversity metrics, and predictive modeling all together provides a framework to understand the microbial role in tumor identification.

### 5.1 Data preprocessing

Data preprocessing is a crucial first step in building AI‐driven decision‐making systems for microbiome‐based cancer prediction. Proper preprocessing can greatly improve the performance and generalizability of ML models.^149^, ^151^ Some major challenges in microbiome data analysis are issues such as varying sequencing depths, compositional effects, and outliers. Therefore, data transformation and normalization techniques are needed. Normalization methods aim to rescale features to a standard range so that features with larger magnitudes do not dominate the learning process. Common methods are L1 and L2 normalization.^152^, ^153^ In addition, it has been demonstrated that combining normalized data with original taxonomic profiles can further improve the performance of classification algorithms.^149^ Another common approach is to apply logarithmic transformations, e.g. *log*10 or natural logarithm, to reduce the influence of dominant features and mitigate the impact of outliers.^151^, ^154^ When dealing with varying sequencing depths and data sparsity, Bayesian transformations like those used in hybrid microbiome network inference approaches can be an effective technique.^144^

In general, microbiome data are high dimensional, that is, more features than samples. High‐dimensional inputs typically lead to challenges such as model overfitting and higher computational cost.^155^ Hence, feature selection and dimensional reduction methods are crucial preprocessing steps. Simple statistical tests such as the *t*‐test and Mann–Whitney *U* test can be leveraged to rank features based on their discriminatory power. However, these methods are not ideal and may end up selecting redundant or correlated features.^156^ Moreover, methods like recursive feature elimination evaluate subsets of features by training a model on each subset and assessing its performance. The embedded methods perform feature selection during model training. For example, *L*1 regularization adds a penalty to the model's loss function that encourages sparsity in the feature weights, effectively performing feature selection. Similarly, tree‐based models like random forest and gradient boosting machines can rank features based on their importance. Moreover, techniques such as minimum redundancy–maximum relevance and linear discriminant analysis effect size are commonly utilized in microbiome studies for feature selection and preprocessing purposes.^53^, ^157^, ^158^, ^159^, ^160^ Furthermore, methods like principal component analysis have been leveraged to simplify complex microbiome data sets and reduce their dimensionality.^107^, ^161^ Finally, the choice of optimal preprocessing depends on the data set characteristics, feature correlation, sparsity, and the nature of the downstream analysis.

### 5.2 Model design

Choosing the right ML model architecture is a critical step in AI‐driven microbiome analysis and impacts its performance, computational cost, and ability to generalize to new data. Researchers have used a diverse range of learning‐based algorithms for microbiome data analysis. This review provides an overview of the most common architectures along with their applications and trade‐offs. Readers interested in detailed architectural implementation specifics are encouraged to explore the referenced studies.

#### 5.2.1 Logistic regression

Logistic regression is a linear statistical model that has been widely used in microbiome research for various tasks. For example, researchers have benefited from logistic regressions for both feature selection and prediction of host phenotypes associated with cancer development and progression.^154^, ^162^ One of the main advantages of logistic regression is its great interpretability. The linear decision boundary of the model allows quantifying each feature's contribution toward the prediction outcome. For example, Topçuoglu et al.^163^ leveraged logistic regression models to find and characterize statistically significant correlations between specific bacterial biomarkers and various types of cancer. While logistic regression offers great interpretability, studies have shown that this linear model performs poorer than other methods such as naïve Bayes or more complex nonlinear models like artificial neural networks.^164^ It might be due to complex nonlinear relationships between input and output in high‐dimensional classification scenarios in microbiome analysis. Furthermore, these models have been widely utilized in other medical domains, ranging from cancer research^165^ to dental implant survival prediction.^166^

#### 5.2.2 Random Forests

Random Forest (RF) model is a powerful and widely adopted ensemble learning method, renowned for its robustness and high predictive accuracy across diverse ML tasks, including classification and regression.^167^ At its core, RF operates by constructing a multitude of individual decision trees during the training phase and combining their predictions to produce a more stable and accurate overall output. This is achieved through two key randomization techniques. First, each tree in the forest is trained on a distinct bootstrap sample of the original training data, a process known as bagging. This ensures that each tree is exposed to a slightly different subset of the data, promoting diversity among the individual learners.^168^ Second, when splitting a node within each tree, only a random subset of features is considered, rather than evaluating all available features. This “feature bagging” further decorrelates the individual trees, preventing any single dominant feature from dictating the structure of all trees and thereby reducing the variance of the ensemble. For classification, the final prediction is determined by a majority vote among the trees, while for regression, it is the average of their predictions. The inherent design of Random Forests offers several significant advantages, making them particularly suitable for complex, high‐dimensional data sets common in scientific research. Their ensemble nature inherently mitigates the risk of overfitting, a common challenge with single decision trees, by averaging out the noise and errors of individual, unpruned trees.^168^, ^169^ Furthermore, RF models can effectively handle both numerical and categorical features, are robust to outliers and missing data without requiring extensive preprocessing, and can intrinsically provide estimates of feature importance. This latter capability, often derived from metrics like Gini impurity reduction or permutation importance, allows researchers to identify the most influential variables in their predictive models. Despite their interpretability as black‐box models being less direct than individual decision trees, their superior predictive performance and built‐in diagnostic tools often outweigh this limitation, positioning Random Forests as a foundational algorithm in contemporary data‐driven analyses.^170^

#### 5.2.3 *k*‐Nearest Neighbors

*K*‐Nearest Neighbors (KNNs) are a nonparametric, instance‐based learning algorithm suitable for both classification and regression tasks. Given a data set as a set of points in a high‐dimensional feature space, the KNN algorithm classifies or predicts the value of a new instance based on the characteristics of its KNNs. The neighbors are determined by a preselected distance metric, which measures the dissimilarity or similarity between samples. The common distance metrics used in KNN are Euclidean distance, Manhattan distance, Minkowski distance, cosine similarity, and correlation coefficients. In classification tasks, the class label of the new sample is determined by a majority vote among the observed class labels of its *k* nearest neighbors, that is, the most frequent class among the neighbors. Whereas in regression tasks, the prediction for the new sample is the average or median of target values of its *k* nearest neighbors, that is, the average or median of the neighboring values. KNNs have been used in various tasks in the medical domain, such as microbiome‐based cancer research,^159^ identifying cancer using microbial community profiles of salivary flora,^124^ or dental caries classification from x‐ray images.^171^

#### 5.2.4 Naïve Bayes classifiers

The Naïve Bayes classifier is a probabilistic ML model designed based on the fundamental basics of Bayes' theorem. It is a principled way of computing the posterior probability of a class given a set of features (see the Appendix S1 for a mathematical representation). The *naïve* part of this algorithm comes from its assumption of conditional independence between the features. Although this conditional independence assumption is often violated in real‐world biological data, where many features are correlated, the Naïve Bayes classifier has performed surprisingly well and achieves robust performance. It is particularly effective in capturing dominant patterns and relationships within the high‐dimensional feature spaces while remaining computationally efficient.

There are different variants of Naïve Bayes classifier, each designed for a specific type of data. For continuous feature data, there is Gaussian Naïve Bayes which assumes features within each class are drawn from a normal, that is, Gaussian distribution. For discrete feature data, especially those with count data or frequency information, for example, word counts in text analysis or read counts from microbiome sequencing data, Multinomial Naïve Bayes is used. For binary feature data, for example, presence or absence data, Bernoulli Naïve Bayes is the suitable choice. In the context of microbiome analysis, Multinomial Naïve Bayes has been great for taxonomic classification of 16S rRNA gene sequences from complex microbial communities. This is further improved by region‐specific training where the classifier is trained on data from specific anatomical locations or environmental niches that increase taxonomic resolution and allow for more precise identification of microbial taxa in the samples being analyzed. Naïve Bayes classifiers have become popular and widely used in various tasks such as bioinformatics^125^ and oral and dental disease diagnosis.^172^, ^173^

#### 5.2.5 Support vector machines

Support vector machines (SVMs) are a robust and flexible class of supervised ML algorithms for both classification and regression tasks. Fundamentally, SVMs work by constructing a hyperplane, or a set of hyperplanes in the case of multi‐class classification, in a high‐dimensional feature space that partitions the data points into distinct and well‐separated classes. The linear SVM tries to find the best hyperplane that provides the maximum margin between classes. This hyperplane can be specified as *w* · *x* + *b* = 0, where *w* is the weight vector orthogonal to the hyperplane that defines its orientation and *b* is the bias term that is the offset from the origin. The margin is the distance between the hyperplane and the nearest data points from each class, and its size is 2/∥*w*∥. For data sets not linearly separable in the original *d*‐dimensional feature space, SVMs use kernel functions. These kernel functions implicitly map the data points into a higher‐dimensional feature space, often of infinite dimensionality, where linear separation is possible. The radial basis function kernel is one of the popular and effective choices among various kernel functions since it can capture complex nonlinear relationships in the data.^174^

SVMs have been widely applied in the medical data analysis field, ranging from inferior alveolar nerve injury identification in dentistry^175^ to many bioinformatics tasks including cancer biomarker identification using microbiome data.^107^, ^124^, ^125^, ^163^ Though designed for binary classification, SVMs can be extended to multiclass classification by one‐versus‐all or one‐versus‐one techniques. In the one‐versus‐all approach, a binary SVM is trained for each class against all other classes. However, it has been found that applying SVM to multi‐cancer prediction using microbiome data often leads to suboptimal performance and high computational cost, especially when dealing with high‐dimensional microbiome data.^159^ These issues are due to problems such as class imbalance, where some classes have much fewer samples compared to other classes, and high feature dimensionality, which may lead to overfitting. Another problem is that training a multiclass SVM classifier is computationally expensive and its complexity increases drastically with the size of the data set. Thus, computation‐efficient alternatives or preprocessing techniques may be necessary.

#### 5.2.6 Artificial neural networks

Artificial neural networks (ANNs) are nonlinear function approximators inspired by the structure of biological neural networks. These networks are typically comprised of three or more layers of processing units, called neurons or nodes, arranged in an input layer, one or more hidden layers, and an output layer. The input layer gets the external data, denoted as a vector *x* = (*x*_1_, *x*_2_, …, *x*_*n*_) where *n* is the number of input features. Each neuron in a hidden layer connects to neurons in the previous layer and combines their outputs with adjustable weights and a bias term. This combined value then passes through a nonlinear activation function, for example, sigmoid or ReLU, that allows the network to recognize complex relationships in the data.

Training an ANN means updating these weights and biases to minimize prediction errors. Prediction errors are typically measured in terms of loss functions. For example, mean squared error or cross‐entropy loss functions are commonly used for regression or classification tasks. This process is achieved through a gradient‐based backpropagation mechanism. By working backward through the network, it calculates how much each weight and bias contribute to the overall error.

Then, optimization algorithms, such as stochastic gradient descent, leverage this information to update the value of those weights and biases to minimize the error rate. Please see the Appendix S1 for a detailed explanation of ANN algorithms. This complex interaction of interconnected neurons and nonlinear activation functions makes ANN's decision‐making process a *black box*. While deep neural networks are very good at learning complex patterns and extracting features from raw data, it is much harder to understand how they achieve specific prediction outcomes.^176^ Methods are being developed to make these models more interpretable; for example, attention mechanisms^177^ allow the network to focus on parts of the input data that drive the model prediction. Such approaches try to provide some visualization about the state of the network.^178^ However, this remains an open research area in explainable AI algorithms. ANNs, due to their unique characteristics in learning complex nonlinear patterns, have been employed in a wide range of tasks, such as virtual drug discovery,^179^ cancer research,^180^ and oral and dental health.^181^

### 5.3 Evaluation techniques

#### 5.3.1 Model interpretability

The model's design and performance are tied to the data characteristics, that is, diversity, quality, quantity, and the broader research context. Figure 4 shows a comparison of different ML architectures to guide this selection process. A key consideration in model selection is the trade‐off between interpretability and performance.^182^ In applications like tumor profiling where understanding the relationships between microbiome features and cancer characteristics is important, interpretable models have a great advantage. Models like logistic regression and decision trees provide transparency into these relationships. Therefore, researchers can see which features drive the model predictions.^183^ This transparency comes from interpretable coefficients in logistic regression models or explicit decision rules in decision trees. However, in tasks like cancer screening where predictive performance is more important, more complex models like artificial neural networks and boosting algorithms may be preferred. Such models, while often performing better, are conceived as *black boxes*, making it hard to understand the underlying mechanisms driving their predictions. In scenarios where performance and interpretability are both important, RF models are often a good choice. They balance between performance and interpretability through feature importance and individual tree analysis.^158^, ^159^, ^184^

Computational cost is another important factor to consider. ANNs and SVMs, especially with complex architectures, are generally more computationally expensive than simpler models like logistic regression and decision trees. This means longer training times and potentially more hardware. For example, SVMs scale with the number of support vectors, which can be substantial for large data sets. Deeper neural networks with several hidden layers and interconnected neurons require significant computational resources for gradient‐based optimization during training. Moreover, data availability also plays a big role in determining which modeling approaches are feasible. The limited availability of large and well‐curated microbiome data sets makes simpler models like logistic regressions or SVMs a more suitable choice, rather than data‐hungry deep learning models like ANNs in cancer‐related microbiome studies.^107^ Deep learning models need huge amounts of data to learn complex patterns and avoid overfitting. In microbiome research, where sample sizes can be limited due to logistical or financial constraints, simpler models that generalize well from smaller data sets are often preferred. RFs, with their ensemble nature and inherent robustness to overfitting, and SVMs, with their ability to handle high‐dimensional data even with limited sample sizes, are most commonly used in this field.^53^, ^73^, ^106^, ^107^, ^121^, ^124^, ^125^, ^185^

#### 5.3.2 Data leakage

An essential concern throughout the analytical pipeline is preventing the contamination of sensitive data from feature engineering and transformation through model training and even evaluation. Data leakage refers to the inadvertent use of validation or test set information in the training phase, leading to inflated performance assessment metrics and misleading assessments of model efficacy.^186^ This inflation is due to the fact that the model *learned* information that, in real scenarios, it would not have access to. Thus, it severely compromises its generalizability to unseen data. There are different types of data leakage, including target leakage, train‐test contamination, and cross‐validation leakage. These are especially common for scaling or normalization, feature selection, and dimensionality reduction, which have parameters or transformations computed on the entire data set and then on the train‐test split. This would introduce information from the test set into the training process, violating the fundamental core principle of keeping validation data separate and leading to overly optimistic performance estimates.^187^, ^188^, ^189^

Any lapse in data leakage has considerable consequences since it yields unreliable performance metrics, which do not correspond to the model's true performance. Consequently, this could lead to the selection of suboptimal models or instill misplaced confidence in the applicability of a model in the real world.^188^, ^190^ For example, if the data are scaled before splitting into training and test sets, this means the scaling parameters, such as mean, variance, etc., are influenced by the test set, effectively leaking information. In cross‐validation, applying preprocessing before creating folds allows information from the held‐out folds to influence the training data. Rather, the ideal approach should be to perform and complete all preprocessing steps within each cross‐validation fold or after splitting the data, ensuring that only training data is used for transformation and model training.^187^

#### 5.3.3 Evaluation and validation

Rigorous model performance and generalizability assessment mandates a held‐out independent test data set. Test data should be strictly separated from the training data and are considered the ultimate arbiter of whether a model will generalize to unseen real‐world data. Cross‐validation techniques, especially *k*‐fold cross‐validation with appropriate stratification, are also necessary to avoid overfitting and improve model reliability. *k*‐fold cross‐validation splits the data into *k* equal or approximately equal folds or portions. Then, the model is trained *k* times. During each training, *k* – 1 folds are used to train, and the remaining fold is leveraged to validate the model. In this way, it effectively simulates multiple train‐test splits, hence providing a more robust and statistically sound estimate of model performance compared to a single train‐test split.^191^ Results for any given iteration are then averaged to get an overall measure of the model performance. Furthermore, patient‐level stratification is suggested to ensure that the characteristics of patients are represented in all folds, thus eliminating the possibility of introducing biases and further improving the robustness of cross‐validation outcomes. Ultimately, hyperparameter tuning is important for peak performance and efficiency.^125^ Hyperparameters, which are parameters that are not learned during the training process but rather set before training, significantly influence the model's learning behavior and final performance. The process of hyperparameter optimization involves systematically searching the hyperparameter space to identify the combination that yields the best model performance on a validation set. Several search strategies exist, ranging from basic methods like grid search, which exhaustively evaluates all possible combinations within a predefined grid, to random search, which samples random combinations from the hyperparameter space, to more sophisticated approaches.^179^ These more advanced methods are often more computationally efficient, especially when dealing with high‐dimensional hyperparameter spaces, and can lead to better performing models than grid or random search.^124^

#### 5.3.4 Performance metrics

Measuring the performance of a learning‐based algorithm requires established metrics that capture different aspects of its predictive ability. There are several different metrics that provide a complete picture of models' performance. For example, the accuracy rate is a fundamental metric that quantifies the overall correctness of the model's predictions by calculating the proportion of correctly predicted instances out of the total number of samples. While accuracy is intuitive, it can be misleading, especially in imbalanced data sets where one class is much larger than the others. In such cases, a model can achieve high accuracy by just predicting the majority class most of the time. To address such limitations, F1 score is often used.^192^ F1 score is the harmonic mean of precision and recall. Precision measures the proportion of true positive predictions out of all positive predictions, that is, how well the model avoids false positives. Recall, on the other hand, measures the proportion of true positive predictions out of all actual positive instances, that is, how well the model avoids false negatives. Therefore, F1 score is a very useful metric when false positives and false negatives have big consequences. Another metric to evaluate a model's performance is the area under the receiver operating characteristic curve (AUC). The receiver operating characteristic (ROC) curve plots the true positive rate, that is, recall, against the false positive rate at various classification thresholds. AUC is the area under this curve and measures the model's ability to distinguish between positive and negative instances across all possible thresholds. AUC is widely used and is a helpful metric as it does not get affected by class imbalance and provides a global measure of the model's discriminative power.^73^, ^124^, ^125^

### 5.4 Applications of AI in oral microbiome analysis

Several recent studies have investigated the use of ML methods to uncover the complex relationship between the oral microbiome and cancer, with a particular emphasis on OSCC. These studies, as listed in Table 3, use different ML models and data sets to find diagnostic biomarkers and see how model architecture affects the outcome. For instance, He et al.^125^ investigated the predictive potential of oral microbiome signatures for OSCC risk using four ML classifiers, including random forest, support vector machines, artificial neural networks, and naive Bayes. Their findings demonstrated the superior performance of the RF model, achieving a significantly higher predictive rate compared to the other classifiers. Indeed, after adjustment for confounders, an optimized RF model using 20 specific bacterial genera achieved an excellent AUC of 0.99 and surpassed the microbial dysbiosis index in performance, with the advantages of targeted microbial biomarkers shown in risk prediction.

**TABLE 3:** Summary of key studies investigating the use of ML to investigate the relationship between the oral microbiome in HNSCC and OSCC.

Author (year)	Task	Collected sites	Model	Performance	Class size
Warnke‐Sommer et al. (2017)^107^	OSCC classification	OSCC lesions	Support vector machines	AUC: 1.0	OSCC: 19, Healthy: 19
Zhou et al. (2020)^73^	OSCC classification	OSCC lesions	Random forest	AUC: 0.82	OSCC: 24
Zhou et al. (2021)^121^	OSCC classification	Saliva, subgingival plaque, tumor surface, healthy mucosa, and tumor tissue	Random forest	ACC: 98.17%	OSCC: 47, Healthy: 48
Eun et al. (2021)^106^	LN metastasis identification	Saliva	Random forest	AUC: 0.89	LN: 20, No LN: 34
Banavar et al. (2021)^118^	Oral cancer diagnosis	Saliva	Logistic regression	AUC: 0.9, Sensitivity: 83%, Specificity: 97%	Oral cancer: 71, Healthy: 171
Saxena et al. (2022)^53^	OSCC classification	Swab samples	Random forest		OSCC: 34, Healthy: 32
Banavar et al. (2023)^123^	OSCC and OPSCC	Saliva	Logistic regression	Specificity: 94%, Sensitivity: 90%	Positive: 92, Negative: 805
Freitas et al. (2023)^185^	Cancer type classification	TCMA cancer specific	Random forest	ACC: 90%	HNSCC: 155, STAD: 127, COAD: 125, ESCA: 60, READ: 45
Meng et al. (2023)^124^	ESCC classification	Saliva	Random forest, Gaussian mixture, KNN, logistic regression, SVM, XGBoost	Accuracy: 89%, Precision: 89%, Recall: 90%, F1‐score: 90%, AUC: 0.97	ESCC: 4500, Control: 4500
He et al. (2024)^125^	OSCC classification	Saliva	Random forest, support vector machines, artificial neural networks, and naïve Bayes	AUC: 0.99	OSCC: 80, Healthy: 17
Praveen et al. (2024)^91^	Oral cancer diagnosis	Saliva	Light Gradient Boosting Machine	Accuracy: 97%, precision: 98%, Sensitivity: 96%, F1 score: 90%, AUC: 0.98	Oral cancer: 157, Control: 865

Abbreviations: AUC, area under the curve; ESCC, esophageal squamous cell carcinoma; KNN, *k*‐nearest neighbors; LN, lymph node; OC, oral cancer; OPSCC, oropharyngeal squamous cell carcinoma; OSCC, oral squamous cell carcinoma; SVM, support vector machine; TCMA, Cancer Microbiome Atlas.

Zhou et al.^121^ presented a noninvasive approach for OSCC diagnosis using oral microbiota profiling from five different sites, including saliva, subgingival plaque, tumoral surfaces, healthy mucosa, and tumoral tissue. They developed two random forest models: one using all five sites and the other using saliva only. The complete random forest model obtained an accuracy of 98.17% while the one using only saliva obtained an accuracy of 95.70%. They validated their findings using external cross‐validation data sets yielding results of 96.67% and 93.58%, respectively. This study is significant as it demonstrates great diagnostic potential while incorporating minimally invasive approaches, such as salivary microbiome profiling. Besides, they report some genera of bacteria associated with OSCC along with their possible mechanisms of pathogenesis, including *Actinobacteria*, *Fusobacterium*, *Moraxella*, *Bacillus*, and *Veillonella*. Another study by Zhou et al.^73^ presents an RF model that examined the relationship between OSCC and the microbiome via sequencing the 16S rRNA gene in cancerous lesions and the associated noncancerous tissues of 24 OSCC patients. It uncovered functional changes to the microbiome and recognized an alteration of amino acid metabolism and glucose consumption, both of which are known for being dysregulated in OSCC. The RF classifier indicated a 12 bacterial‐genus signature to distinguish cancerous tissues from paracancerous tissues, for which AUC was 0.82. This shows the diagnostic potential in clinical practice using tissue‐level information. Compared to the study by Zhou et al.,^121^ this study looks at tissue‐specific changes and functional implications rather than a broad diagnostic model.

In another interesting study, Saxena et al.^53^ investigated the impact of smokeless tobacco on the oral microbiome and its association with OSCC using an RF model. They analyzed a total of 196 oral microbiome samples collected from healthy subjects and OSCC patients from three different oral sites. They made key observations about the covariates affecting microbiome composition, including health status, sampling site, and smokeless tobacco. The results showed that the oral microbiomes of smokeless tobacco users who were healthy were similar to those of OSCC patients. Thus, smokeless tobacco might promote the growth of inflammation‐associated microbial species. This highlights the importance of considering environmental factors like tobacco use in microbiome‐based cancer studies. They found a potential link between tobacco use, microbiome changes, and OSCC by associating certain metabolites with microbial genera, including *Prevotella*, *Capnocytophaga*, and *Fusobacterium*. Moreover, Eun et al.^106^ explored a novel biomarker of LN metastasis in OSCC via oral microbiome signatures. They collected saliva from 54 patients with OSCC and, training a random forest classifier, identified marked differences in microbial diversity between patients with and without LN metastasis. They found that the metastatic group was enriched in *Prevotella* and *Bifidobacterium*, while pathways related to signal peptidase II were more abundant in patients without metastasis.

Banavar et al.^118^, ^123^ conducted two studies for early cancer detection using saliva and logistic regression modeling. In their first study, they developed a diagnostic tool for oral premalignant disorders and early‐stage oral cancer using metatranscriptomic data. They developed a logistic regression model with both taxonomic and functional microbiome features.^118^ This model offered an AUC of 0.9 with 92.3% sensitivity and 97.9% specificity for stage I cancer patients. In their second study, they designed and tested an improved saliva‐based test for the early detection of oral and oropharyngeal cancer.^123^ In this work, they trained the model using 945 saliva samples and evaluated it using 230 independent samples. This model achieved 94% specificity and 90% sensitivity for OSCC and 84.2% in OPSCC. These studies represented saliva as a noninvasive diagnostic medium for early detection of cancer. Moreover, their study showed the value of including human mRNA markers along with microbial ones.

Freitas et al.^185^ studied the use of microbiome data for classifying multiple types of cancer by leveraging tissue‐specific microbial profiles. Using random forest algorithms trained on The Cancer Microbiome Atlas data, they tested the microbiome's ability to classify five different cancer types, including head and neck, colon, stomach, esophagus, and rectum. Their results showed that distinguishing cancers of adjacent sites are difficult due to similar microbial profiles. However, they were able to achieve a good performance for head and neck, stomach, and colon cancers, with over a 90% accuracy rate for colon cancer. This shows the promise and limitations of using microbiome data for pan‐cancer classification and the strong effect of anatomical sites on microbial composition.

Meng et al.^124^ explored the relationship between salivary microbiota and esophageal squamous cell carcinoma (ESCC) using six different ML algorithms, including random forest, Gaussian mixture, KNN, logistic regression, SVM, and XGBoost models optimized by a genetic algorithm. Their results showed the XGBoost model outperformed other models with an AUC of 0.97. These results demonstrated the relative abundance of certain bacteria, for example, *Bacteroides* and *Actinobacteria*, can be used as a diagnostic tool. Praveen et al.^91^ analyzed 1022 saliva samples, 157 from oral cancer patients and 865 from healthy controls, using a light gradient boosting machine model to find bacterial genera associated with oral cancer. The model identified *Streptococcus*, *Parvimonas*, *Corynebacterium*, and *Prevotella* as significant factors. Specifically, their results showed oral cancer patients had higher *Streptococcus* and *Parvimonas*, which correlated with higher pro‐inflammatory cytokines, that is, IL‐6 and TNF‐α and fatty acid oxidation enzymes such as CPT1A and poorer survival outcomes. Conversely, higher *Prevotella* and *Corynebacterium* were associated with better survival outcomes. These results show that specific changes in the oral microbiome, along with changes in cytokines and fatty acid metabolism, can be predictive markers for oral cancer. Moreover, Warnke‐Sommer et al.^107^ developed a framework based on the SVM algorithm for early detection of oral carcinoma using oral microbiome biomarkers. To optimize the SVM training, they used several preprocessing steps, for example, centering, scaling, and PCA to reduce the dimension of raw microbiome data. To test the model on unseen data, they used 10‐fold cross‐validation, which is a robust method to evaluate model performance and generalizability. Their SVM model trained on bacterial abundances achieved an AUC of 1.0. This means the model has very high discriminatory power and can accurately classify individuals with and without oral carcinoma based on their oral microbiome.

## 6 LIMITATION AND FUTURE DIRECTIONS

The integration of ML algorithms with oral microbiome research has already significantly advanced our understanding of OSCC. At the same time, several limitations still remain. The limitations could be summarized into three major groups: study design and data characteristics, model interpretability and validation, and biological context. The heterogeneity in study design concerning sample size, patient demographics, sampling site, and data‐processing methods are critical obstacles. Indeed, the heterogeneity limits the comparison of different studies and the generalization of their findings. Future studies will be necessary, including larger, well‐powered multicenter investigations with standardized protocols relative to sample collection, processing, and sequencing. Studies also need to focus on more diverse populations so the models can perform well across ethnic and geographic variations. While RF models have been used very frequently with great success,^53^, ^73^, ^106^, ^121^, ^125^, ^185^ other advanced ML techniques such as deep learning and graph neural networks should be explored. In this regard, the success of XGBoost by Meng et al.^124^ suggests that more complex algorithms can be used. Furthermore, the performance of different ML algorithms needs to be tested in a systematic way using well‐curated benchmark data sets. Such studies will provide better insights to choose a suitable modeling approach based on the research question and data characteristics. Moreover, additional interdisciplinary research is needed to address the interpretability of ML models' decision‐making process in microbiome research. This limitation will be facilitated by explanatory ML models or the incorporation of explainability techniques to elucidate how the predictions are being made. This would reduce variability and increase reproducibility through standard operating protocols of sample collection, processing, and data analysis. External validation by independent cohorts will confirm the strengths of models with applicability in real‐world clinical settings.

In addition, factors such as diet, oral hygiene, and tobacco use affect the oral microbiome, but many studies have not considered these variables and therefore may be confounded. Although some taxonomic associations have been identified, the functional implications of microbiome alteration have barely been explored, which limits the understanding of underlying biological mechanisms. Sampling site and data types are also a challenge; saliva is minimally invasive but does not capture the whole tumor microenvironment, as noted by Zhou et al.^121^ and Banavar et al.^118^, ^123^ Multimodal studies, where different sample types such as saliva, tissue, and plaque, along with multi‐omics approaches, for example, metagenomics and metatranscriptomics, are integrated, will become necessary for a more complete understanding. The use of metatranscriptomic data from Banavar et al.,^118^ including human mRNA, highlights the value of this integration, while Praveen et al.^91^ demonstrated the importance of linking microbiome changes with cytokines and metabolic pathways.

Mainstream studies are cross‐sectional, precluding the ability to address the causal relationship between microbiome alterations and cancer progression. Understanding the temporal involvement of microbiome changes in OSCC development will thus require longitudinal studies. This will also enhance the predictive value of microbiome signatures toward early detection and risk stratification. Therefore, integrating microbiome data with other omics data, such as genomics and transcriptomics, would potentially provide an overview of OSCC pathogenesis and thus enable the identification of multifaceted biomarkers. Further focusing on functional aspects of microbiome alterations, such as metabolic pathways and host–immune interactions, may uncover mechanistic insights and therapeutic targets. Future studies should address the concerns about lifestyle and environmental factors including dietary issues, oral hygiene, and alcohol or tobacco consumption to separate (or integrate) the effect of these factors from disease‐specific microbiome changes. Besides, translating the ML models into clinical applications requires rigorous validation through independent and large‐scale clinical trials. Researchers should consider developing standardized diagnostic and prognostic tests to validate the findings from microbiome data and how to integrate these data into clinical settings. Some previous studies discussed the use of cross‐validation for the microbial signatures.^107^ However, external validations would be required for clinical translation. In conclusion, while the reviewed studies showcase the potential of ML in microbiome‐based cancer research, overcoming limitations through larger, diverse cohorts, advanced ML techniques, multi‐omics integration, longitudinal studies, and robust clinical validation is vital to fully integrate this approach for enhancing cancer diagnosis, prognosis, and treatment in the clinical setting.

Translation of the ML‐based findings into clinically applicable tools requires independent and large‐scale clinical validation. Further studies should focus on the establishment of standardized diagnostic and prognostic tests based on previously validated microbiome signatures and their routine application in clinical practice. More work is needed on the standardization of data, assay reproducibility, and regulatory and ethical issues concerning the use of AI applications in clinical settings. Besides rigorous validations using cross‐validation, external corroboration is critical for clinical applications.^107^ In summary, though the studies reviewed herein demonstrated a great potential for ML in microbiome‐based cancer research, the need for addressing limitations by using larger and more diverse cohorts, more sophisticated ML techniques, integration of multiomics, longitudinal studies, and strong clinical validation is paramount for the full integration of learning‐based approaches in cancer diagnosis, prognosis, and treatment in clinical settings.

## 7 CLINICAL RELEVANCE

The training approaches designed for diagnostics have the capacity to substantially enhance patient care and drive progress in dental and medical research. This includes the potential to identify individuals at high risk for medical conditions such as disease relapse or progression to a different state. Recently, ML algorithms have demonstrated success in predicting skin cancer prognosis with accuracy comparable to that of experienced dermatologists.^193^, ^194^ Machine learning has the potential to enhance oncology care, and numerous studies already have explored its applications in cancer risk assessment, diagnosis, drug development, and molecular tumor profiling by analyzing pathology reports and imaging data. When combined with oral microbiome analysis, AI has greater potential to combat OSCC. AI detects particular microbial patterns linked to cancer; thus, it provides a noninvasive and cost‐effective point for early diagnosis, risk assessment, and individualized treatment plan creation. Early detection is crucial for OSCC since, mostly, it is diagnosed at a late stage, thus affecting the survival rate and quality of life of the affected individuals. For instance, one such fact is that stage IV OSCC accounts for most cancer deaths, and posttreatment survival is noted to progress up to 80–90% if detected in stage I or II.^195^ The AI tools can analyze very complicated microbiome data to better predict how the illness is progressing, enabling the healthcare provider to make better decisions at the right time for timely treatment.

That being said, many challenges accompany the integration of these innovations into day‐to‐day clinical practice, such as the establishment of standardized mechanisms to guarantee uniformity in sample acquisition, sequencing, and analysis, and the inclusion of diverse patient populations to make findings generalizable. Most studies associating AI with periodontology are based on images, demographic data, and clinical findings.^196^, ^197^ The field is growing, and with the incorporation of analysis of microbiome data to monitor periodontitis,^198^ OSCC detection can advance faster (e.g., multidisease oral omics‐based biomarkers). The bias of models is possible due to the underreporting of demographic information, symptoms, etc., when combined with diagnostic parameters for AI to analyze such as radiologic data, imaging, liquid biopsies, or microbiome data sets. There are implications in the variability that can occur, leading to poor training of the model. Also, long‐term, large‐scale studies are needed to validate the technological efficiency of AI microbiome models, as some small‐scale studies will not carry out the same results once the data becomes larger. What lies ahead are numerous subjective and objective benefits, and once these challenges are tackled, AI will lead to drastic change in the management of oral cancer through early intervention with overall reduced incidences and mortality. Another major benefit would be the long‐term monitoring of patients to detect relapse. Microbiome follow up via AI would certainly be a noninvasive way to monitor setbacks. If the mentioned barriers are overcome, the integration of AI and microbiome analysis could pave the way for groundwork in precision oncology, thus presenting new opportunities for preventing, more extensively diagnosing, and then subsequently treating OSCC.

## 8 CONCLUSION

OSCC remains a serious health burden worldwide with relatively high rates of morbidity and mortality due to late diagnosis and limited treatment options. The intersection of microbiome research and AI has emerged as a strong paradigm toward the advancement of early detection strategies, refining prognostication, and developing personalized therapeutic interventions tailored according to the particular profile of a patient in the management of OSCC. This review offers a comprehensive analysis of disruptions in the oral microbiota, as shown in a growing body of evidence. We focus on specific alterations in microbial community composition, structure, and functional potential. These changes are associated with shifts in metabolic pathways and gene expression profiles, contributing to the risk of OSCC initiation and its subsequent stagewise progression. AI‐driven analyses, leveraging advanced ML models capable of handling high‐dimensional and complex biological data, have yielded critical insights into these distinct microbial signatures, facilitating the development of accurate and noninvasive diagnostic and prognostic tools with potential clinical translation. These findings emphasize the transformational role that AI may play in unlocking the inherent complexity of oral microbiome data toward the identification of novel microbial biomarkers with diagnostic, prognostic, or therapeutic implications. These therapeutic biomarkers will contribute to more effective personalized clinical management strategies for OSCC.

However, translating such findings into actual clinical benefits would be tangible only when several key areas are pursued in future investigations. These include (1) the rigorous standardization of experimental and computational methodologies, encompassing sample collection, DNA/RNA extraction, sequencing protocols, bioinformatic analysis pipelines, and ML model training and evaluation procedures, to ensure reproducibility, comparability, and generalizability of findings across independent studies and diverse populations; (2) the expansion of cohort diversity to include individuals from diverse demographic backgrounds, geographic locations, and lifestyle factors, thereby enhancing the generalizability of findings and mitigating potential biases; and (3) the implementation of well‐designed longitudinal studies with appropriate statistical power and rigorous controls to establish definitive causal relationships between specific microbial alterations and OSCC development, progression, and response to therapy. Furthermore, AI will help researchers rigorously validate reported findings in independent and well‐characterized cohorts. By fostering and strengthening interdisciplinary collaborations among microbiome researchers, AI specialists, clinical oncologists, and other relevant disciplines, the field can accelerate progress toward achieving the overarching goal of precise, predictive, and preventive care strategies for oral cancer patients worldwide. This can ultimately improve patient outcomes, reduce the global burden of this devastating disease, and pave the way for personalized medicine in oncology.

## FUNDING INFORMATION

This work was supported by the funds provided by the National Institutes of Health, grants CA233037 and DE030427 (A.P.) and the University of North Carolina at Chapel Hill.

## CONFLICT OF INTEREST STATEMENT

The authors declare no conflicts of interest.

## ETHICS STATEMENT

NA.

## PATIENT CONSENT STATEMENT

NA.

## PERMISSION TO REPRODUCE MATERIAL FROM OTHER SOURCES

NA.

## CLINICAL TRIAL REGISTRATION

NA.

## Supporting information


Appendix S1.

## Data Availability

Data sharing not applicable to this article as no data sets were generated or analyzed during the current study.
