# Supplementary material for: Recent advancements in artificial intelligence‐powered cancer prediction from oral microbiome
Source: Periodontol 2000. 2025 Sep 11;98(1):181–213. doi: 10.1111/prd.70000 (PMC12842876; doi:10.1111/prd.70000)
Supplement: Supplementary file 1 — Appendix S1. [file PRD-98-181-s001.docx]

**Appendix**

## **MeSH Terms used for search in the PubMed database.**

The following terms in 5 different categories were used to generate the search queries using “AND” and “OR” operators.

| **Category (term count)** | **Term** |
| --- | --- |
| **Oral Cancer (17)** | - Mouth Neoplasm - Neoplasm, Mouth - Neoplasms, Mouth - Neoplasms, Oral - Neoplasm, Oral - Oral Neoplasm - Oral Neoplasms - Cancer of Mouth - Mouth Cancers - Mouth Cancer - Cancer, Mouth - Cancers, Mouth - Oral Cancer - Cancer, Oral - Cancers, Oral - Oral Cancers - Cancer of the Mouth |
| **Oral squamous cell carcinoma (17)** | - HNSCC - Head And Neck Squamous Cell Carcinomas - Carcinoma, Squamous Cell of Head and Neck - Squamous Cell Carcinoma of the Head and Neck - Head and Neck Squamous Cell Carcinoma - Squamous Cell Carcinoma, Head And Neck - Oral Squamous Cell Carcinoma - Squamous Cell Carcinoma of the Mouth - Oral Cavity Squamous Cell Carcinoma - Oral Squamous Cell Carcinomas - Squamous Cell Carcinoma of the Larynx - Squamous Cell Carcinoma of Larynx - Laryngeal Squamous Cell Carcinoma - Squamous Cell Carcinoma of the Nasal Cavity - Oropharyngeal Squamous Cell Carcinoma - Hypopharyngeal Squamous Cell Carcinoma - Oral Tongue Squamous Cell Carcinoma |
| **Head and Neck cancer (33)** | - Head and Neck Neoplasm - Head, Neck Neoplasms - Neoplasms, Head and Neck - Head Neoplasms - Head Neoplasm - Neoplasm, Head - Neoplasms, Head - Neck Neoplasms - Neck Neoplasm - Neoplasm, Neck - Neoplasms, Neck - Cancer of Head and Neck - Cancer of the Head and Neck - Head and Neck Cancer - Upper Aerodigestive Tract Neoplasms - Upper Aerodigestive Tract Neoplasm - UADT Neoplasm - Neoplasms, UADT - Neoplasm, UADT - Neoplasms, Upper Aerodigestive Tract - UADT Neoplasms - Cancer of Neck - Neck Cancers - Cancer of the Neck - Neck Cancer - Cancer, Neck - Cancers, Neck - Cancer of Head - Head Cancers - Cancer of the Head - Head Cancer - Cancer, Head - Cancers, Head |
| **Microbiome (16)** | - Microbiotas - Microbial Community - Community, Microbial - Microbial Communities - Microbial Community Composition - Community Composition, Microbial - Composition, Microbial Community - Microbial Community Compositions - Microbiome - Microbiomes - Human Microbiome - Human Microbiomes - Microbiome, Human - Microbial Community Structure - Community Structure, Microbial - Microbial Community Structures |
| **Artificial intelligence (19)** | - Intelligence, Artificial - Computer Reasoning - Reasoning, Computer - AI (Artificial Intelligence) - Machine Intelligence - Intelligence, Machine - Computational Intelligence - Intelligence, Computational - Computer Vision Systems - Computer Vision System - System, Computer Vision - Systems, Computer Vision - Vision System, Computer - Vision Systems, Computer - Knowledge Acquisition (Computer) - Acquisition, Knowledge (Computer) - Knowledge Representation (Computer) - Knowledge Representations (Computer) - Representation, Knowledge (Computer) |

# **Naïve Bayes Classifiers**

The Naïve Bayes classifier is a probabilistic machine learning model based on the fundamental basics of Bayes theorem. It's a principled way of computing the posterior probability of a class $C$ given a set of features $X$. Bayes theorem is the mathematical foundation of this classifier, which is:

$$P(C\mid X)=\frac{P(X\mid C)\cdot P(C)}{P(X)}$$

Here $P(C\mid X)$ is the posterior probability of class $C$ given the features $X,P(X\mid C)$ is the likelihood of observing the feature vector $X$ given the data point comes from class $C,P(C)$ is the prior probability of class $C$ before we see the features, and $P(X)$ is the probability of observing the feature vector $X$, which is a normalization constant to make the posterior probabilities sum to 1 . The naïve part of this classifier comes from its assumption of conditional independence between the features, which is mathematically written as:

$$P(X\mid C)=\prod_{i=1}^{n} P\left( x_{i}\mid C \right)$$

where $x_{i}$ is the $i$-th feature and $n$ is the total number of features in the feature vector $X$. Although this conditional independence assumption is often violated in real-world biological data where many features are correlated, the Naïve Bayes classifier has been found to be surprisingly robust. In high-dimensional feature space, it captures the dominant patterns and relationships in the data and is computationally efficient.

The type of data being analyzed requires a specific variant of the Naïve Bayes classifier. For continuous features data, there is Gaussian Naïve Bayes, which assumes features within each class are drawn from a normal, i.e., Gaussian distribution. For discrete feature data, especially those with count data or frequency information, such as word counts in text analysis or read counts from microbiome sequencing data, Multinomial Naïve Bayes is used. For binary feature data, e.g., presence or absence data, Bernoulli Naïve Bayes is the way to go. In the context of microbiome analysis, Multinomial Naïve Bayes has been great for the taxonomic classification of 16S rRNA gene sequences from complex microbial communities. This is further improved by region-specific training where the classifier is trained on data from specific anatomical locations or environmental niches that increase taxonomic resolution and allow for more precise identification of microbial taxa in the samples being analyzed. However, since biological datasets often violate the conditional independence assumption, Naïve Bayes classifiers have become popular and widely used in bioinformatics even for big and complex microbiome datasets [125].

## **Artificial Neural Networks**

Artificial neural networks (ANNs) are non-linear function approximators inspired by the structure of biological neural networks. These networks are made up of layers of processing units, called neurons or nodes, arranged in an input layer, one or more hidden layers, and an output layer. The input layer gets the external data, denoted as a vector $\mathbf{x}=\left( x_{1},x_{2},\ldots,x_{n} \right)$ where $n$ is the number of input features. The hidden layers perform non-linear transformations of this data. Each neuron in layer $l$ gets input from the neurons in the previous layer $(l-1)$. The output of a neuron $j$ in layer $l$ can be written as:

$$a_{j}^{(l)}=\sigma\left( \sum_{i=1}^{m} w_{ji}^{(l)}a_{i}^{(l-1)}+b_{j}^{(l)} \right)$$

where $a_{j}^{(l)}$ is the output of neuron $j$ in layer $l,\sigma$ is the activation function, e.g., sigmoid, tanh or ReLU, $w_{ji}^{(l)}$ is the weight connecting neuron $i$ in layer $(l-1)$ to neuron $j$ in layer $l,a_{i}^{(l-1)}$ is the activation of neuron $i$ in layer $(l-1),b_{j}^{(l)}$ is the bias term for neuron $j$ in layer $l$, and $m$ is the number of neurons in layer $(l-1)$. These weights $\left( w_{ji}^{(l)} \right)$ and biases $\left( b_{j}^{(l)} \right)$ are the model's primary parameters, which are learned during the training process.

Training an ANN means updating the weights and biases so that some loss function, $L(y,y)$, is minimized where $y$ is the network predictions and $y$ is the true target values. The usual loss functions are Mean Squared Error (MSE) for regression and Cross-Entropy Loss for classification. Backpropagation is a gradient-based approach that makes smart use of the chain rule of calculus to compute the gradients of the loss function with respect to every weight and bias. Optimization algorithms take these gradients $\frac{\partial L}{\partial w_{ji}^{(L)}}$ and $\frac{\partial L}{\partial b_{j}^{(L)}}$, and use those to update the parameters to minimize the loss. In practice, we use variants of Stochastic Gradient Descent (SGD), including mini-batch SGD, SGD with momentum, and Adam. The general update rule for a weight is:

$$w_{ji}^{(l)}=w_{ji}^{(l)}-\eta\frac{\partial L}{\partial w_{ji}^{(l)}}$$

where $\eta$ is the learning rate. It's because of this complex interaction of interconnected neurons and non-linear activation functions that ANNs are black boxes. While deep neural networks are very good at learning complex patterns and extracting features from raw data automatically, it's much harder to understand why they make their predictions [125, 199]. Methods are being developed to make these models more interpretable, for example, attention mechanisms [177], which allow the network to focus on parts of the input data, and visualization methods for networks [178] which try to give some visualization about the state of the network. However, this remains an open research area for these models.
